# Fasting-mimicking diet alleviates inflammatory pain by inhibiting neutrophil extracellular traps formation and neuroinflammation in the spinal cord

**DOI:** 10.1186/s12964-023-01258-2

**Published:** 2023-09-21

**Authors:** Ting Li, Ying Yue, Yan Ma, Ziwen Zhong, Miaomiao Guo, Jie Zhang, Zhiping Wang, Changhong Miao

**Affiliations:** 1 Department of Anesthesiology, Zhongshan Hospital, Fudan University; Cancer Center, Zhongshan Hospital, Fudan University, Shanghai, 200032 China; 2 Shanghai Key Laboratory of Perioperative Stress and Protection, Shanghai, China; 3 Department of Anesthesiology, The Affiliated Hospital of Xuzhou Medical University, Xuzhou, China

**Keywords:** Inflammatory pain, Neutrophil extracellular traps, Fasting-mimicking diet, Neuroinflammation, MAO

## Abstract

**Background:**

Neutrophil extracellular traps (NETs) promote neuroinflammation and, thus, central nervous system (CNS) disease progression. However, it remains unclear whether CNS-associated NETs affect pain outcomes. A fasting-mimicking diet (FMD) alleviates neurological disorders by attenuating neuroinflammation and promoting nerve regeneration. Hence, in this study, we explore the role of NETs in the CNS during acute pain and investigate the role of FMD in inhibiting NETs and relieving pain.

**Methods:**

The inflammatory pain model was established by injecting complete Freund’s adjuvant (CFA) into the hind paw of mice. The FMD diet regimen was performed during the perioperative period. PAD4 siRNA or CI-amidine (PAD4 inhibitor) was used to inhibit the formation of NETs. Monoamine oxidase-B (MAO-B) knockdown occurred by AAV-GFAP-shRNA or AAV-hSyn-shRNA or was inhibited by selegiline (an MAO-B inhibitor). The changes in NETs, neuroinflammation, and related signaling pathways were examined by western blot, immunofluorescence, ELISA, and flow cytometry.

**Results:**

In the acute phase of inflammatory pain, NETs accumulate in the spinal cords of mice. This is associated with exacerbated neuroinflammation. Meanwhile, inhibition of NETs formation alleviates allodynia and neuroinflammation in CFA mice. FMD inhibits NETs production and alleviates inflammatory pain, which is enhanced by treatment with the NETs inhibitor CI-amidine, and reversed by treatment with the NETs inducer phorbol 12-myristate 13-acetate (PMA). Mechanistically, the neutrophil-recruiting pathway MAO-B/5-hydroxyindoleacetic acid (5-HIAA) / G-protein-coupled receptor 35 (GPR35) and NETs-inducing pathway MAO-B/ Reactive oxygen species (ROS) are significantly upregulated during the development of inflammatory pain. MAO-B is largely expressed in astrocytes and neurons in the spinal cords of CFA mice. However, knockdown or inhibition of MAO-B effectively attenuates CFA-induced inflammatory pain, NETs formation, and neuroinflammation in the spinal cord. Moreover, within rescue experiments, MAO-B inhibitors synergistically enhance FMD-induced pain relief, NETs inhibition, and neuroinflammation attenuation, whereas supplementation with MAO-B downstream molecules (i.e., 5-HIAA and PMA) abolished this effect.

**Conclusions:**

Neutrophil-released NETs in the spinal cord contribute to pain development. FMD inhibits NETs formation and NETs-induced neuroinflammation by inhibiting the MAO-B/5-HIAA/GPR35 and MAO-B/ROS pathways in astrocytes and neurons, thereby relieving pain progression.

Video Abstract

**Supplementary Information:**

The online version contains supplementary material available at 10.1186/s12964-023-01258-2.

## Background

Acute pain is normal but prone to transform into intractable persistent pain [1, 2]. In fact, a significant proportion of patients (ranging from 20 to 56%) undergo transition from acute postoperative pain to chronic pain, despite the active use of available medications [3]. Persistent neuroinflammation triggered by trauma is the major contributing factor to the initiation and sensitization of pain [4]. Timely and adequate treatment of neuroinflammation during acute pain reduces the risk of turning into chronic pain [2].

Emerging evidence has shown that the intricate bidirectional communication between immune cells infiltrating the central nervous system (CNS) and CNS resident cells, such as neurons and glial cells, plays a crucial role in pain progression [4, 5]. In neuropathic and inflammatory pain models, neutrophils infiltrating the CNS cause neuroinflammation and central sensitization by releasing proinflammatory mediators [4]. In addition, activated neutrophils release neutrophil extracellular traps (NETs) which are web-like structures composed of double-stranded DNA, histones, and granule proteins, such as neutrophil elastase and myeloperoxidase (MPO) [6, 7]. These NETs are involved in traumatic brain injury, Alzheimer's disease, and stroke [8–10], with neuroinflammation and neurotoxicity identified as the primary mechanisms through which NETs exacerbate these neurological disorders [11, 12]. However, it is unclear whether NETs in the CNS affect pain outcomes by regulating neuroinflammation.

Fasting-mimicking diet (FMD) is a periodic feeding strategy based on a diet that provides limited calories, low sugar, low protein, and high unsaturated fat to minimize the burden of fasting and offer adequate micronutrients [13, 14]. Growing evidence indicates that different forms of dietary restrictions effectively limit the progression of various chronic diseases such as diabetes mellitus, cancer, and neurological disorders [14–16]. In particular, FMD increases synaptic plasticity and neurogenesis and promotes axonal regeneration and recovery after sciatic nerve injury [17]. Evidence from a clinical trial suggests that a modified fasting regimen with a cycle of 7–21 days is effective in treating chronic pain syndrome [18]. Nevertheless, the molecular mechanisms underlying the pain-reducing effects of fasting remain largely elusive.

Therefore, in this study, we aim to explore the potential analgesic effects of FMD through the inhibition of neutrophil extracellular traps (NETs) and NETs-induced neuroinflammation, and further elucidate the role of spinal cord NETs in inducing pain, with the goal of providing novel insights for postoperative pain prevention and treatment.

## Methods

### Animals and surgery

Experiments were conducted with approval from the Experimental Animal Care and Use Committee of Zhongshan Hospital, Fudan University (Shanghai, China). Male C57 adult mice (6–8 weeks old) were sourced from Shanghai Jihui Laboratory Animal Care Center (Shanghai, China) and housed in a specific pathogen-free environment at the Department of Laboratory Animal Science, Fudan University. Mice in the complete Freund's adjuvant (CFA) model group were anesthetized with isoflurane before injecting CFA (20 μL, Sigma, St. Louis, MO) in the right hind paw to induce inflammatory pain. The mice in the sham-operated group received an injection of an equivalent volume of saline solution.

### Mouse diet strategy

The mice in the normal diet group were fed AIN-93G purified rod chow containing 4 kcal/g of gross energy, with calories supplied by proteins, carbohydrates, and fat in a percentage ratio of 20:64:16. The mice in the FMD group were fed a diet cycle comprising three periods (day 1, days 2–4, and days 5–7), as described previously [19, 20]. Day 1 diet was provided at 50% of the normal daily caloric intake (50% of AIN-93G intake, 0.11 kJ/g carbohydrates, 1.17 kJ/g protein, and 0.53 kJ/g fat). The diet provided on days 2–4 was 10% of the normal daily caloric intake (10% of AIN-93G intake, 0.34 kJ/g carbohydrates, and 0.01 kJ/g protein). On days 5–7, mice were fed a normal diet (AIN-93G), followed by a new round of FMD. Two FMD cycles were performed one week before and one week after CFA administration for a total of 14 days. The body weight of each mouse was monitored daily; weight losses did not exceed 20%.

### Assessment of pain behaviors

The pain threshold was measured on the day before CFA injection as the baseline value. The mice were tested for pain behavior at 9:00 a.m. on the 1^st^, 3^rd^, 5^th^, and 7^th^ day after CFA injection. The behavioral pain test was performed by an investigator blinded to the experimental conditions. To examine mechanical allodynia, we used the up-and-down method to record the paw withdrawal threshold (PWT) [21]. For thermal hyperalgesia, the Hargreaves method was used, as described previously [22]. The paw withdrawal latency (PWL) was determined as the time from the beginning of radiant heat application to mouse paw withdrawal. To prevent paw damage, a cutoff time of 20 s was implemented. Each mouse underwent three tests, with at least 5 min between each test.

### Evans blue staining

Each mouse was injected with 200 μL of 2% Evans blue dye (#E2129, Sigma-Aldrich, USA) through the tail vein; 3 h later, ice-cold phosphate-buffered saline (PBS) was injected into the heart to remove the dye from the blood vessels. During profound anesthesia, the spinal cord segments of the mice were removed, homogenized, and placed in 0.5 mL of a formamide solution to dissolve the Evans blue stain. After incubating at 55 ℃ for 48 h, the sample was centrifuged for 12,000 × g for 30 min at room temperature. The absorbance of the supernatant was determined at 632 nm. The Evans blue content in spinal cord tissue was determined using the standard curve.

### SiRNA screening and intrathecal delivery

Four siRNAs targeting mouse PAD4 mRNA and a scrambled control siRNA were provided by GenePharma (Shanghai, China). The nucleotide sequences of the PAD4 siRNA and scrambled siRNA are listed in Table 1. SiRNA was transfected into PC12 cells with liposome 3000 (Invitrogen, USA). Quantitative polymerase chain reaction and western blotting were used to detect the interference efficiency of PAD4 siRNA. PAD4 siRNA2, 3, and 4 inhibited the expression of PAD4 protein by 68.7%, 54%, and 53.9%, respectively. In vivo analysis revealed that intrathecal injection of PAD4 siRNA4 significantly inhibited PAD4 protein abundance in the spinal cord. Accordingly, the PAD4 siRNA4 was selected for subsequent experiments.

**Table 1:** Sequences (5′-3′) of PAD4 siRNA

Gene	Forward primer	Reverse primer
siRNA1	GGUUCGAGUUUCAUACUAUTT	AUAGUAUGAAACUCGAACCTT
siRNA2	CGCCCAAAGACUUCUUUGATT	UCAAAGAAGUCUUUGGGCGTT
siRNA3	GCACAGCACAGACUUCUAUTT	AUAGAAGUCUGUGCUGUGCTT
siRNA4	GUGCAGGGUUUCUGACAAUTT	AUUGUCAGAAACCCUGCACTT

Intrathecal catheterization was conducted as described previously [21]. In brief, five days before CFA surgery, a PE-10 catheter (OD, 0.6 mm; ID, 0.28 mm) was inserted into the subarachnoid space of mice through the L5–L6 intervertebral space. PAD4 siRNA or scrambled siRNA (5 μg/10 μL) was administered into the subarachnoid space every other day following CFA administration.

### Drugs and drug administration

To inhibit NETs formation, CI-amidine (S8141, Selleck Chemicals, USA) was dissolved in saline and injected intraperitoneally at 10 mg/kg for seven days following CFA injection, as described previously [23, 24]. To inhibit the activity of monoamine oxidase-B (MAO-B), selegiline hydrochloride (HY-14199, MCE, USA) was dissolved in saline and administered intraperitoneally at 10 mg/kg for 7 days after CFA administration [25]. To induce NETs production, phorbol 12-myristate 13-acetate (PMA; P8139, Sigma Aldrich, USA) was dissolved according to the manufacturer’s instructions and injected intrathecally at a dose of 0.1 nmol/mouse every other day following CFA injection 1 h before selegiline hydrochloride administration, based on preliminary studies and previous reports [26]. To supplement 5-hydroxyindoleacetic acid (5-HIAA), 5-HIAA (H8876, Sigma Aldrich, USA) was dissolved in saline and intravenously injected at a dose of 100 μmol/mouse for seven consecutive days after CFA administration 1 h before selegiline hydrochloride administration, as reported previously [27]. Control mice were administrated an equivalent volume of vehicle (saline or dimethyl sulfoxide) via the same route.

### Intrathecal injection of AAV virus

To functionally distinguish the role of MAO-B in astrocytes and MAO-B in neurons in pain, we used RNA interference driven by gfaABC1D (rAAV2/5-GFaABC1D-mCherry-5'miR-30a-shRNA (MAO-B)-3miR-30a-WPREs) or hSyn (rAAV2/9-hSyn-mCherry-5miR-30a-shRNA (MAO-B)-3'miR-30a-WPREs) promoters to down-regulate MAO-B levels in spinal cord astrocytes or neurons. To knock down MAO-B, we selected the shRNA sequence AATCGTAAGATACGATTCTGG to construct an AAV viral vector as reported previously [28]. Scrambled shRNA was the negative control (Brain VTA Technology, Wuhan, China).

After the mice were anesthetized with pentobarbital, a PE10 catheter connected to a Hamilton syringe was introduced into the mice's subarachnoid space. Successful catheter entry into the subarachnoid space was indicated by a sudden tail movement. Subsequently, the virus solution (5 μL, 5 × 10^12^ vg/mL) was slowly injected into the subarachnoid space, and the catheter remained in situ for a minimum of 3 min to prevent drug outflow after injection. The CFA model was established at least three weeks after virus injection.

### Western blotting

The L3-L5 spinal cord segments of mice were harvested under deep anesthesia; homogenized in a mixture of RIPA lysis buffer, phosphatase inhibitor, and PMSF (Beijing Solarbio Science and Technology, China); and then centrifuged for 30 min at 4°C at 12,000 rpm. The protein concentration was measured using a BCA protein analysis kit (Thermo Scientific, USA). After adding the loading buffer, the protein was boiled at 90 °C for 10 min, then separated on 12% acrylamide gels, and subsequently transferred onto polyvinylidene fluoride membranes. Following a 2-h block with 5% bovine serum albumin at room temperature, the membranes were incubated with specific primary antibodies overnight at 4 °C, including rabbit anti-ZO-1 antibody (1:5000, 21,773–1-AP, Proteintech, USA), rabbit anti-occludin antibody (1:5000, 27,260–1-AP, Proteintech, USA), rabbit anti-Ly6G antibody (1:500, ab238132, Abcam, Cambridge, USA), rabbit anti-PAD4 antibody (1:1000, 17,373–1-AP, Proteintech, USA), rabbit anti-histone H3 antibody (1:1000, ab5103, Abcam), mouse anti-MPO antibody (1:2000, 66,177–1-1 g, Proteintech, USA), rabbit anti-MAO-B antibody (1:1000, 12,602–1-AP, Proteintech), rabbit anti-GPR35 antibody (1:1000, NBP2-24,640, Novus Biologicals, USA), and mouse anti-GAPDH antibody (1:5000, AC002, Abclonal, China). Membranes were washed with TBST and incubated with horseradish peroxidase-conjugated goat anti-rabbit antibody (1:5000, S0001, Affinity, China) or goat anti-mouse antibody (1:5000, S0002, Affinity) for 2 h at room temperature. Subsequently, protein visualization was accomplished by employing a SuperLumia ECL Plus HRP substrate kit (SQ201, Shanghai Epizyme Biomedical Technology, China), and image detection was conducted using a Tanon image analysis system (Tanon, China).

### Immunofluorescence

Mice were deeply anesthetized with pentobarbital and intracardially perfused with 0.1 M PBS, followed by 4% cold paraformaldehyde. The L3-L5 spinal cords were then harvested, fixed overnight in 4% paraformaldehyde, and dehydrated overnight in cold 20% and 30% sucrose solutions. The collected spinal cord specimens were cut into 20-μm thick slices using a freezing microtome (CM1900, Leica, Germany). After infiltration with 0.3% Triton X-100 for 15 min, sections were blocked with 5% donkey serum for 1 h at room temperature. For single immunofluorescence staining, the sections were incubated with rabbit anti-PAD4 antibody (1:100,17,373–1-AP, Proteintech, USA), mouse anti-glial fibrillary acidic protein (GFAP) antibody (1:150, #3670, Cell Signaling Technology, Danvers, MA, USA) and goat anti-ionized calcium binding adaptor molecule 1 (IBA1) antibody (1:100, ab5076, Abcam). As described previously [21], the integrated fluorescence intensities of IBA1^+^ microglia and GFAP^+^ astrocytes were calculated using ImageJ software (National Institutes of Health, Bethesda, MD, USA).

Tissue slices were incubated with a combination of rabbit anti-histone H3 (1:100, AB5103, Abcam) and mouse anti-MPO (1:100, 66,177–1-Ig, Proteintech) antibodies to detect NETs. The slices were incubated with a mixture of rabbit anti-MAO-B antibody (1:50, 12,602–1-AP, Proteintech), mouse anti-GFAP antibody (1:150, #3670, Cell Signaling Technology), goat anti-IBA1 antibody (1:100, ab5076, Abcam), and mouse anti-NeuN antibody (1:50, MAB377, Millipore, USA) to detect the cellular localization of MAO-B. The sections were subsequently treated with a secondary antibody mixture, including Alexa Fluor 594-labeled donkey anti-rabbit (1:400), Alexa Fluor 488-labeled donkey anti-goat (1:200), and Alexa Fluor 488-labeled donkey anti-mouse (1:200, all Jackson ImmunoResearch), for 2 h at room temperature. Images were obtained using a fluorescence microscope (BX53, Olympus).

### Enzyme-linked immunosorbent assays (ELISAs)

The L3-L5 spinal cord segments of mice were collected under deep anesthesia, homogenized in a mixture of RIPA lysis buffer, phosphatase inhibitor, and PMSF (Beijing Solarbio Science and Technology, China), and then centrifuged for 15 min at 4°C at 12,000 rpm. Tissue homogenate supernatants were analyzed using mouse interleukin (IL)-1β ELISA kits (PCDBM0158), mouse IL-6 ELISA kits (PCDBM0170), mouse TNF-α ELISA kits (PCDBM0282), and 5-HIAA ELISA kits (PCDBA0103, all PC-Biotech, China) according to the manufacturer’s instructions.

### Reactive oxygen species (ROS) production assay

After anesthesia, mice were intracardially perfused with ice-cold PBS to remove the blood. Next, the L3–L5 spinal cord was dissected to prepare a single-cell suspension. The isolated cells were diluted to 1 × 10^7^ cells/mL and seeded into a 24-well plate. The cells were then incubated with DCFH-DA (10 μM, 50101ES01, Yeasen Biotechnology, China) for 30 min at 37 °C. After washing the cells thrice with serum-free medium, they were suspended in PBS and analyzed by flow cytometry.

### Statistical analyses

All data were presented as the mean ± standard error of the mean (SEM). Two-way repeated-measures analysis of variance (ANOVA) followed by Bonferroni’s post-hoc test was used to analyze pain behavior data. One-way ANOVA was used to analyze western blot, immunofluorescence, ELISA, and ROS assay data. The unpaired Student’s *t*-test was used to analyze the differences between two groups. Differences were considered statistically significant at *P* < 0.05. All statistical analyses were performed using GraphPad Prism 8.0 (GraphPad Software, USA).

## Results

### Blood–spinal cord barrier (BSCB) permeability and neutrophil infiltration increase in the spinal cord of CFA mice

**Fig. 1:**
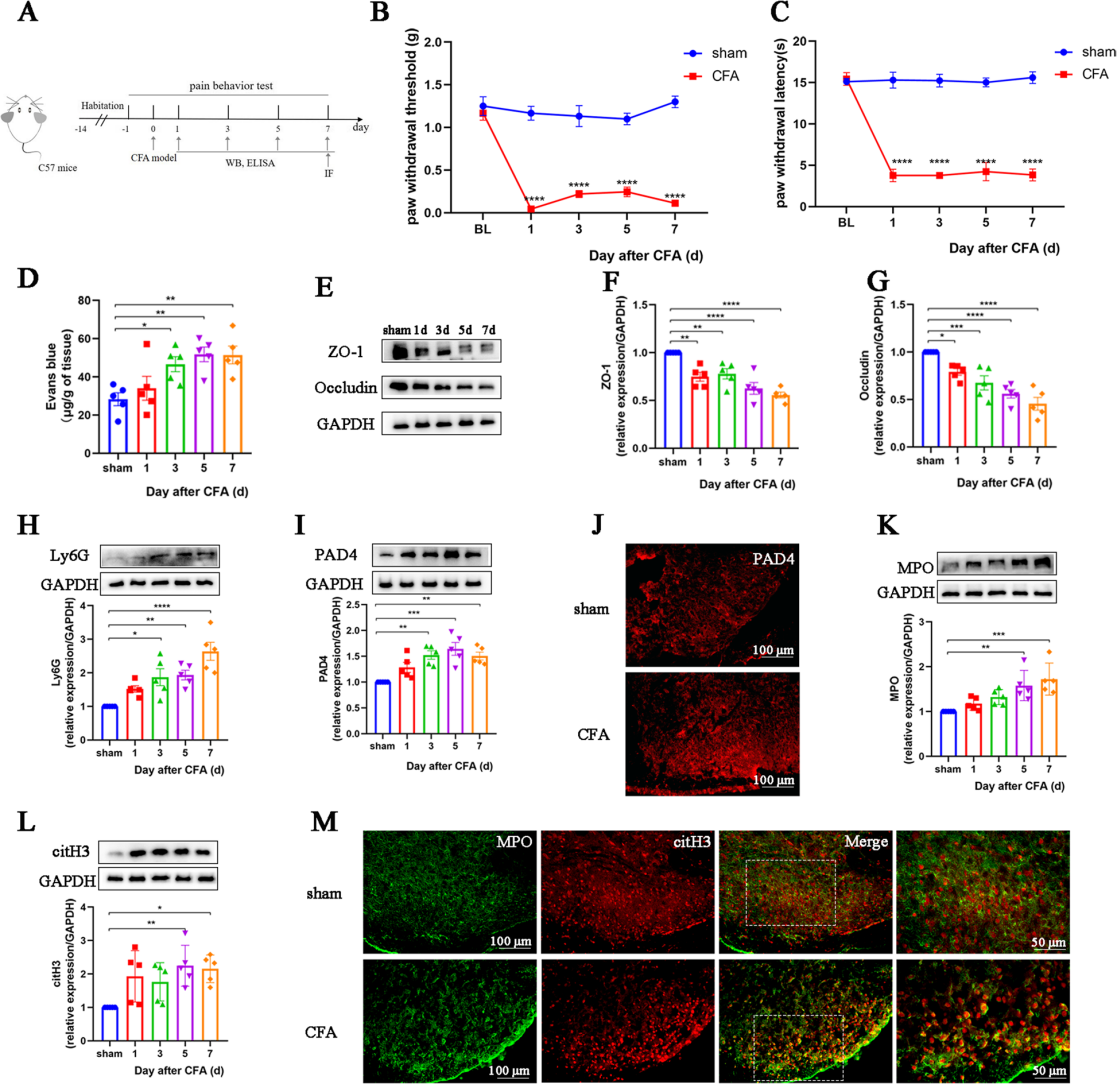
Blood-spinal cord barrier permeability increases and NETs accumulate in the spinal cords of CFA mice. **A**. Timepoint of the schematic design. **B**. Paw withdrawal threshold of mice in each group (*n* = 6, group: F_1, 5 =_ 173.3, *P* < 0.0001; time: F_4, 20 =_ 22.22, *P* < 0.0001; interaction: F_4, 20_ = 21.19, *P* < 0.0001). **C**. Paw withdrawal latency of mice in each group (*n* = 6, group: F_1, 5_ = 2792, *P* < 0.0001; time: F_4, 20_ = 165.5, *P* < 0.0001; interaction: F_4, 20_ = 129.9, *P* < 0.0001). **D**. Quantification of Evans blue dye in the spinal cord of mice (*n* = 5, F_4, 20_ = 5.568, *P* = 0.0035). **E**–**G**. Representative blots and quantification of ZO-1 and occludin in the spinal cord of sham (Day 7) and CFA mice (*n* = 5, ZO-1: F_4, 20_ = 14.74, *P* < 0.0001; occludin: F_4, 20_ = 16.89, *P* < 0.0001). **H** and I. Representative blots and quantification of Ly6G and PAD4 in the spinal cord of sham (Day 7) and CFA mice (*n* = 5, Ly6G: F_4,20_ = 10.96, *P* < 0.0001; PAD4: F_4, 20_ = 8.688, *P* = 0.0003). **J**. Immunofluorescence staining for PAD4 in the spinal cords of sham (Day 7) and CFA (Day 7) mice (*n* = 3, scale bars: 100 μm). **K** and **L**. Representative blots and quantification of MPO, and citH3 in the spinal cord of sham (Day 7) and CFA mice (*n* = 5, MPO: F_4, 20_ = 7.445, *P* = 0.0008; citH3: F_4, 20_ = 4.185, *P* = 0.0127). Ly6G, PAD4, MPO, and citH3 levels in the sham group were set as 1 for quantification. **M**. Double immunofluorescence staining for citH3 (red) and MPO (green) in the spinal cords of sham (Day 7) and CFA (Day 7) mice. NETs were visualized by colocalization of citH3 and MPO staining (merged images; *n* = 3, scale bars: 50 or 100 μm). *P < 0.05, **P < 0.01, ***P < 0.001, ****P < 0.0001, compared with the sham group mice

We first established an inflammatory pain model by injecting CFA unilaterally into the hind paws of mice (Fig. 1A). Behavioral testing suggested that, compared to sham-treated mice, CFA mice exhibited significant mechanical and thermal allodynia on Day 1 post-operation, which lasted until Day 7 of testing (Fig. 1B, C). Then we observed increased neutrophil infiltration in the spinal cords of CFA mice from Day 3 post-operation compared to the sham group (Fig. 1H).

Considering that the BSCB protects against neutrophil infiltration into the spinal cord parenchyma under physiological conditions, we assessed the integrity of the BSCB in CFA mice. The amount of Evans blue stain in the spinal cord of mice increased significantly from Day 3 to Day 7 post-CFA injection (Fig. 1D). Western blot results further revealed that the abundance of tight junction proteins, including ZO-1 and occludin were significantly down-regulated (Fig. 1E–G). This evidence suggests that the impaired BSCB promotes recruitment of circulating neutrophils to the spinal cord during inflammatory pain development.

### NETs accumulate in the spinal cord of CFA mice, along with neuroinflammation

We then detected NETs released by neutrophils in the spinal cord of CFA mice. As shown in Fig. 1I and J, the expression of PAD4, an indispensable enzyme for NETs generation, was dramatically elevated in the spinal cord of CFA-injected mice. Western blot and immunofluorescence further showed that the number of NETs (citH3^+^ MPO^+^) in the spinal dorsal horn of CFA mice was higher than that in the sham group on Days 5 and 7 post-operation (Fig. 1K–M).

**Fig. 2:**
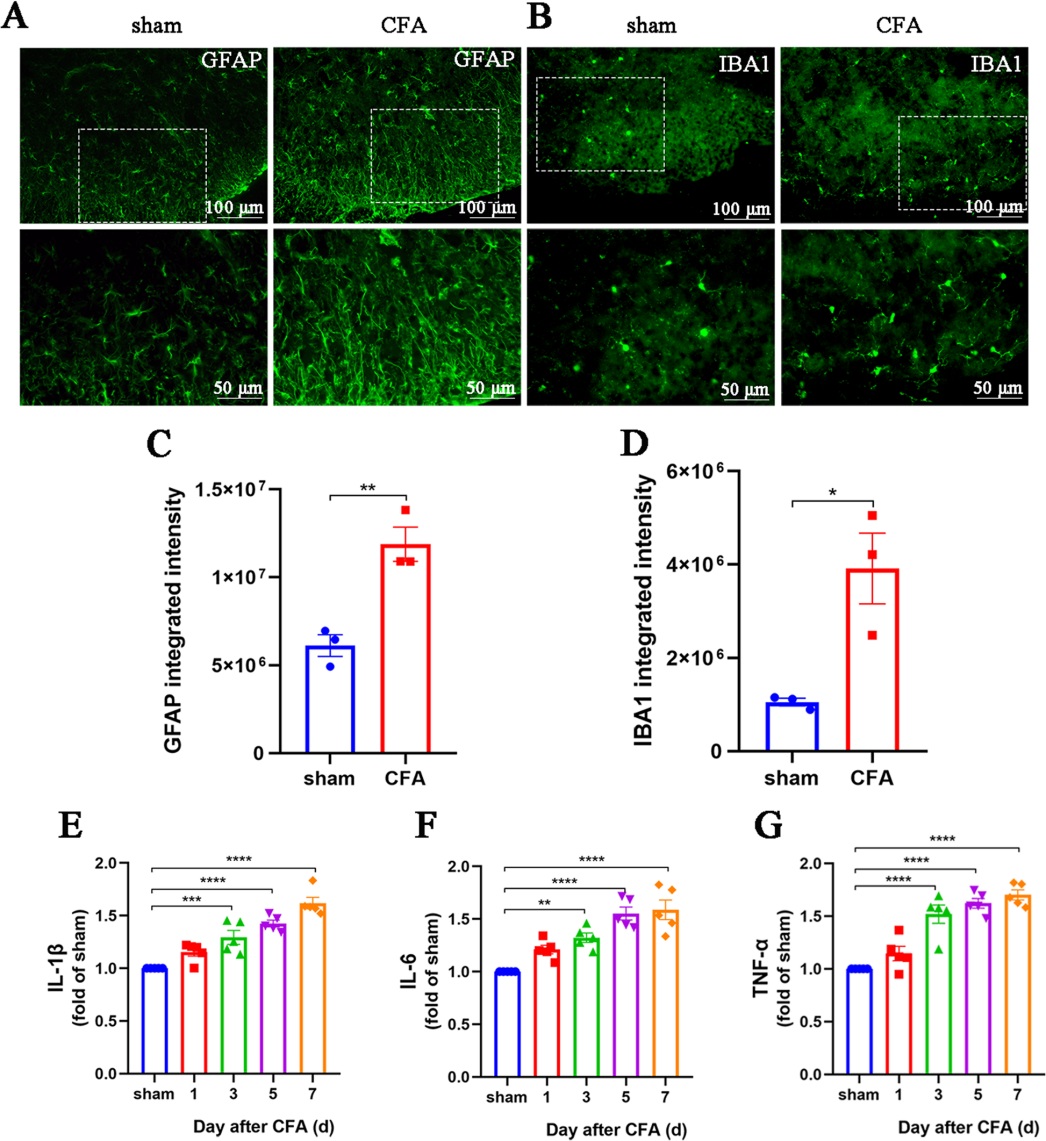
Aggravated neuroinflammation in the spinal cords of CFA mice. **A** and **C**. Representative images and quantification of GFAP^+^ astrocytes in mouse spinal cord sections (*n* = 3, scale bars: 50 or 100 μm). **B** and **D**. Representative images and quantification of IBA1^+^ microglia in mouse spinal cord sections (*n* = 3, scale bars: 50 or 100 μm). **E–G**. ELISAs detecting IL-1β, IL-6, and TNF-α in the spinal cords of mice (*n* = 5, IL-1β: F_4, 20_ = 28.29, *P* < 0.0001; IL-6: F_4, 20_ = 18.35, *P* < 0.0001; TNF-α: F_4, 20_ = 28.91, *P* < 0.0001). Expression in the sham group was set to 1 for quantification purposes. *P < 0.05, **P < 0.01, ***P < 0.001, ****P < 0.0001, compared with the sham group mice

Considering previous studies suggesting that NETs aggravate neuroinflammation [29], we also assessed changes in astrocytes, microglia, and proinflammatory factors within the spinal cords of CFA mice. The number of astrocytes and their branches increased, indicating considerable activation (Fig. 2A, C). Similarly, microglia were activated, characterized by enlargement of the cell body and an increase in their number (Fig. 2B, D). In addition, ELISA results showed that IL-1β, IL-6, and TNF-α levels were notably elevated in the spinal cords of mice injected with CFA (Fig. 2E–G). These findings indicate that NETs are produced in the spinal cords of CFA mice, accompanied by neuroinflammation.

### Inhibition of NETs alleviates allodynia and neuroinflammation in CFA mice

**Fig. 3:**
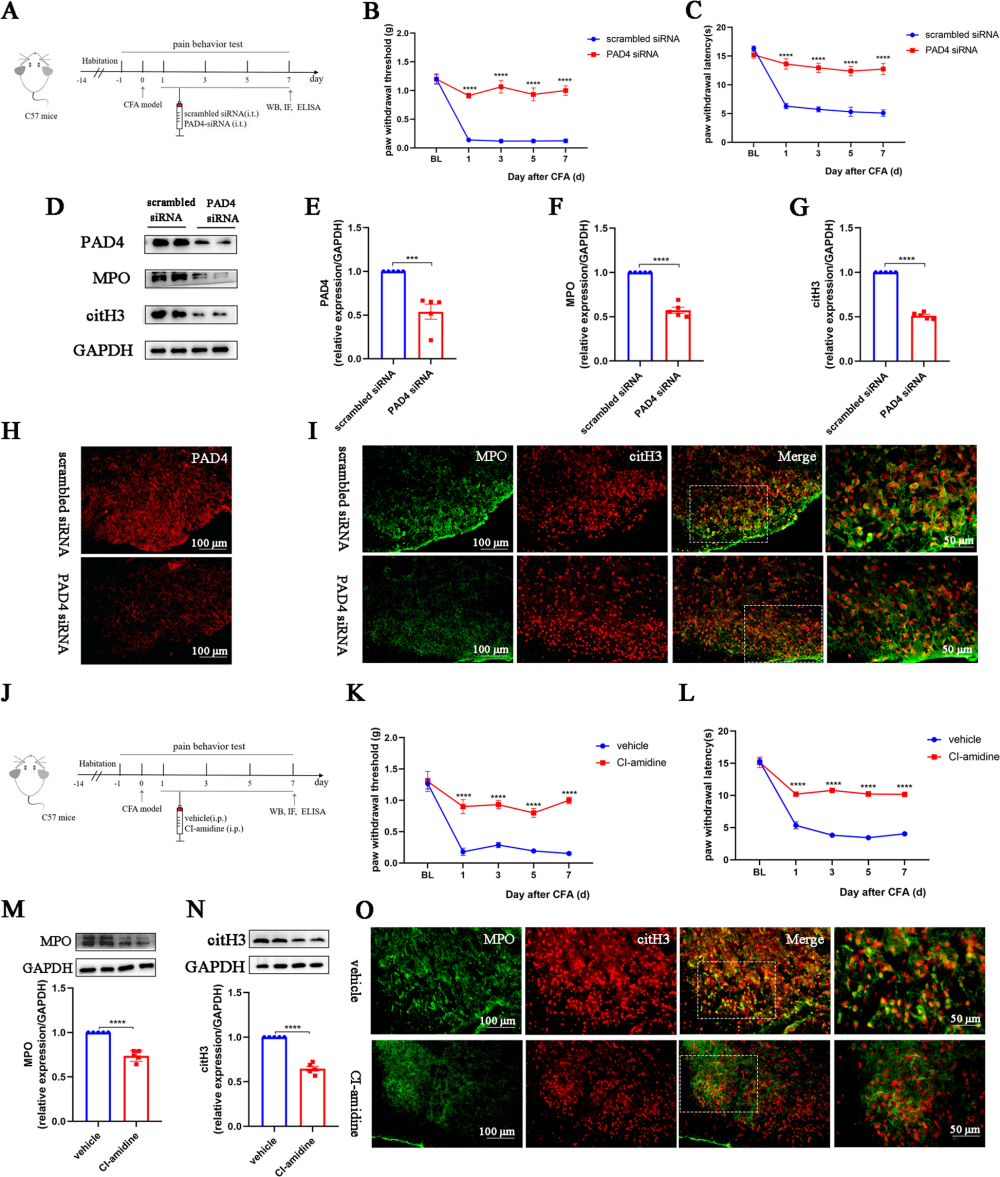
Inhibition of NETs alleviates allodynia in CFA mice. **A**. Schematic of the experimental timeline. **B**. Paw withdrawal threshold of mice in each group (*n* = 6, group: F_1, 5_ = 77.6, *P* = 0.0003; time: F_4, 20_ = 39.98, *P* < 0.0001; interaction: F_4, 20_ = 24.39, *P* < 0.0001). **C**. Paw withdrawal latency of mice in each group (*n* = 6, group: F_1, 5_ = 99.68, *P* = 0.0002; time: F_4, 20_ = 49.51, *P* < 0.0001; interaction: F_4, 20_ = 17.23, *P* < 0.0001). **D–G**. Representative blots and quantification of PAD4, MPO, and citH3 in the spinal cords of scrambled siRNA- and PAD4 siRNA-treated mice (*n* = 5). **H**. Immunofluorescence staining for PAD4 in the spinal cords (*n* = 3, scale bars: 100 μm). **I**. Double immunofluorescence staining for citH3 (red) and MPO (green) in the spinal cords of scrambled siRNA- and PAD4 siRNA-treated mice (*n* = 3). NETs visualized by colocalization of citH3 and MPO staining (merged images; *n* = 3, scale bars: 50 or 100 μm). **J.** Schematic of the experimental timeline.** K**. Paw withdrawal threshold of CFA mice treated with vehicle or CI-amidine (*n* = 6, group: F_1, 5_ = 283.9, *P* < 0.0001; time: F_4, 20_ = 29.81, *P* < 0.0001; interaction: F_4, 20_ = 11.58, *P* < 0.0001).** L**. Paw withdrawal latency of CFA mice treated with vehicle or CI-amidine (*n* = 6, group: F_1, 5_ = 461.2, *P* < 0.0001; time: F_4, 20_ = 100.9, *P* < 0.0001; interaction: F_4, 20_ = 41.12, *P* < 0.0001). **M **and** N**. Representative blots and quantification of MPO, and citH3 in the spinal cords of mice. **O**. Double immunofluorescence staining for citH3 (red) and MPO (green) in the spinal cords of vehicle- and CI-amidine-treated mice (*n* = 3). *P < 0.05, **P < 0.01, ***P < 0.001, ****P < 0.0001, compared with the scrambled siRNA or vehicle group mice

To verify the role of NETs in inflammatory pain, we first intrathecally injected PAD4 siRNA into CFA mice to inhibit NETs generation (Fig. 3A). Compared with the scrambled siRNA group, the mechanical and thermal allodynia of CFA mice in the PAD4 siRNA group were significantly alleviated (Fig. 3B, C). Both western blot and immunofluorescence results verified the successful knockdown of PAD4 in the spinal cord of CFA-treated mice (Fig. 3D, E, and H). Western blot and immunofluorescence revealed significant reductions in MPO and citH3 levels in the spinal cord of CFA mice (Fig. 3D, F, G, I).

**Fig. 4:**
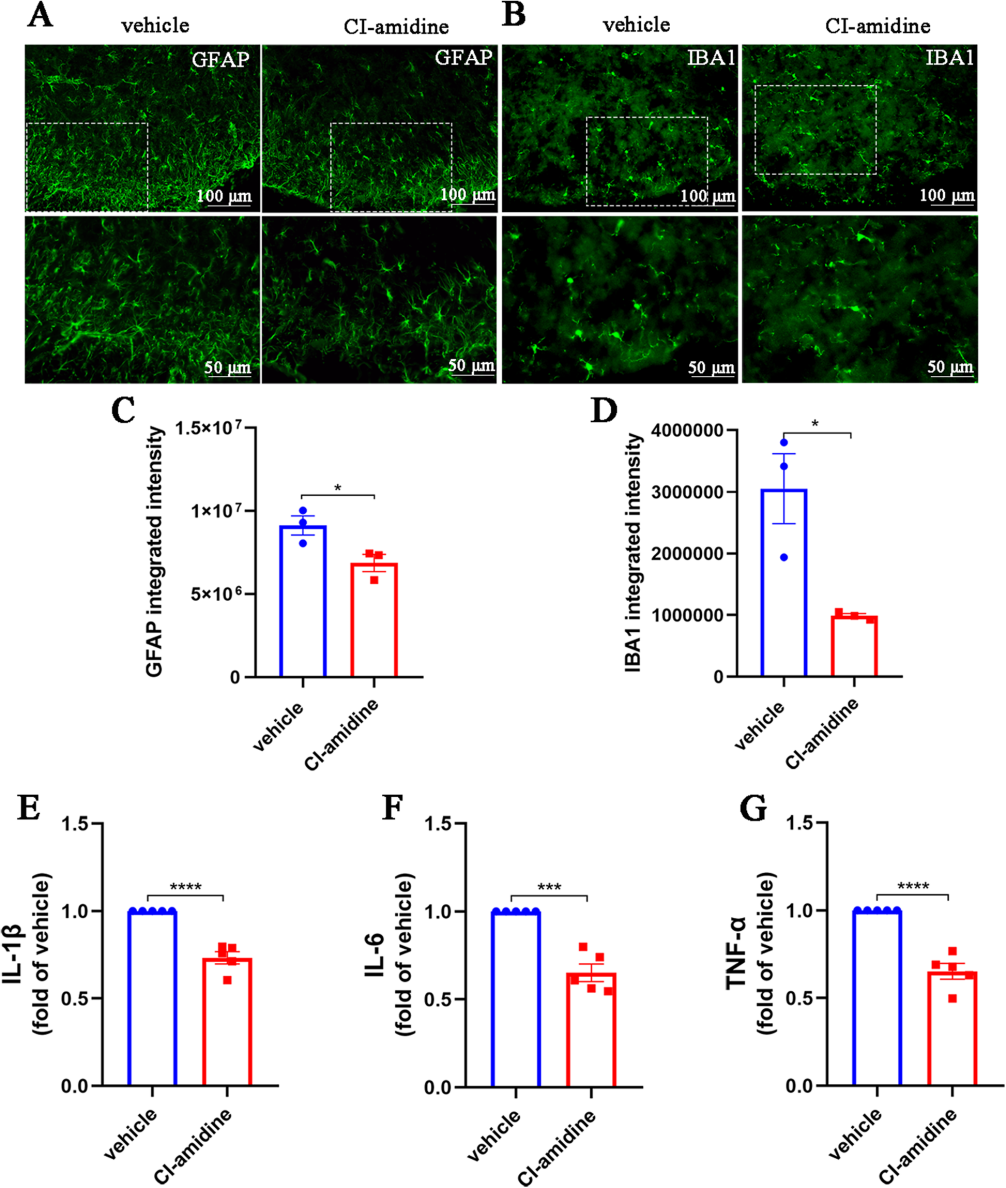
Inhibition of NETs alleviates neuroinflammation of CFA mice. **A** and **C**. Representative images and quantification of GFAP^+^ astrocytes in spinal cord sections (*n* = 3, scale bars: 50 or 100 μm). **B** and **D**. Representative images and quantification of IBA1^+^ microglia in spinal cord sections (*n* = 3, scale bars: 50 or 100 μm). **E–G**. ELISAs detecting IL-1β, IL-6, and TNF-α in the spinal cords of mice (*n* = 5). The expression in the vehicle group was set to 1 for quantification. *P < 0.05, **P < 0.01, ***P < 0.001, ****P < 0.0001, compared with the vehicle group mice

Consistently, CI-amidine, a specific inhibitor of PAD4 (Fig. 3J), alleviated mechanical and thermal allodynia in CFA mice and inhibited NETs formation (Fig. 3K–O). Moreover, CI-amidine treatment reduced the number of activated astrocytes and microglia compared to the vehicle group, along with decreased levels of proinflammatory factors IL-1β, IL-6, and TNF-α (Fig. 4A–G). These results confirm the role of spinal cord neutrophils and NETs in the aggravation of neuroinflammation and inflammatory pain.

### FMD attenuates neuroinflammation and alleviates inflammatory pain by inhibiting NETs formation

**Fig. 5:**
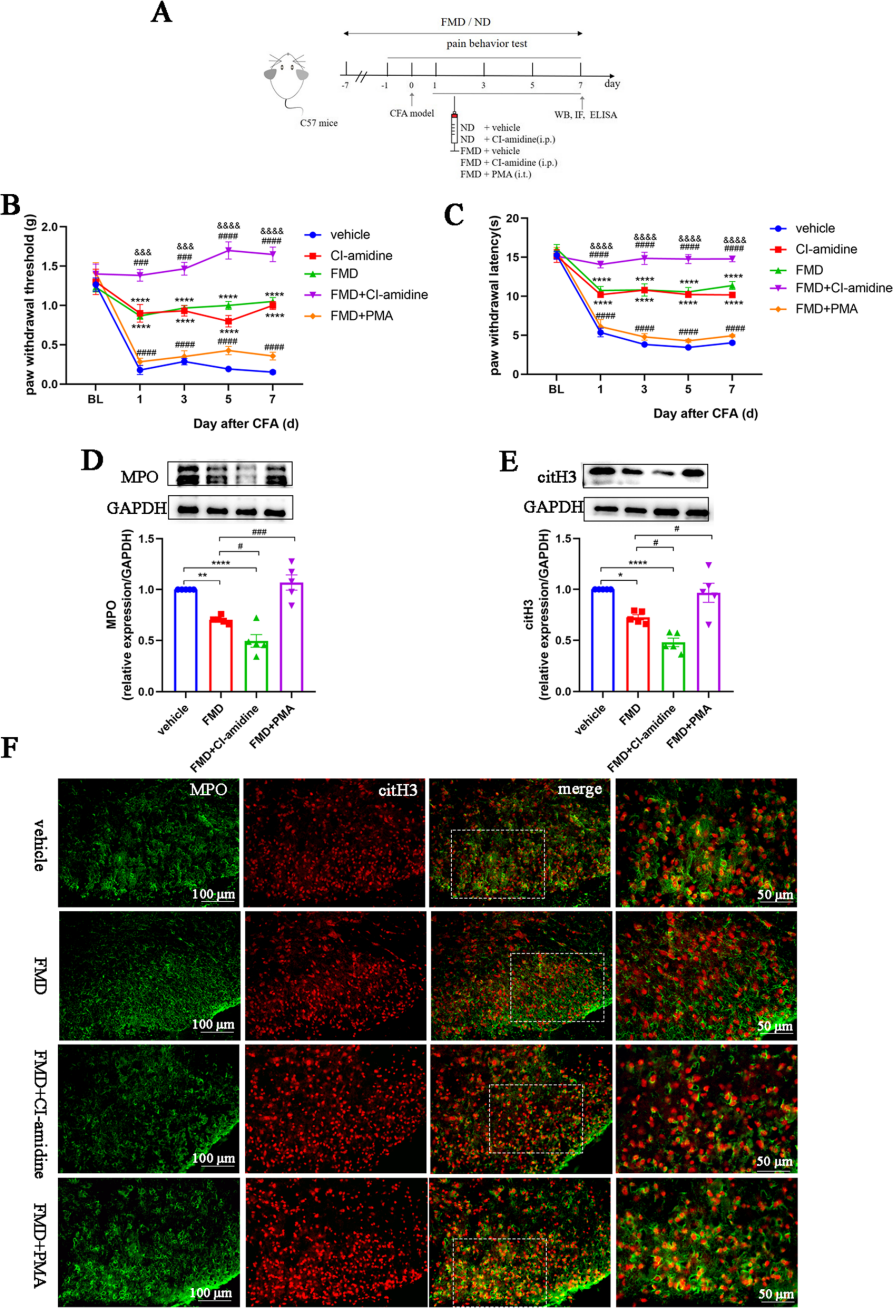
FMD alleviates inflammatory pain by inhibiting the formation of NETs. **A**. Schematic of the experimental timeline. **B**. Paw withdrawal threshold of mice in each group (*n* = 6, group: F_4, 20_ = 93.84, *P* < 0.0001; time: F_4, 20_ = 48.47, *P* < 0.0001; interaction: F_16, 80_ = 11.22, *P* < 0.0001). **C**. Paw withdrawal latency of mice in each group (*n* = 6, F_4, 20_ = 135.1, *P* < 0.0001; time: F (4, 20) = 181.2, *P* < 0.0001; interaction: F (16, 80) = 23.48, *P* < 0.0001). **D** and **E**. Representative blots and quantification of MPO and citH3 in the spinal cords of vehicle, FMD, FMD + CI-amidine, and FMD + PMA groups (*n* = 5, MPO: F (3, 16) = 28.97, *P* < 0.0001; citH3: F (3, 16) = 20.67, *P* < 0.0001). **F**. Double immunofluorescence staining for citH3 (red) and MPO (green) in the spinal cords. NETs visualized by colocalization of citH3 and MPO staining (merged images; *n* = 3, scale bars: 50 or 100 μm). *P < 0.05, **P < 0.01, ***P < 0.001, ****P < 0.0001, compared with the vehicle mice. #*P* < 0.05, compared with the FMD group mice. & *P* < 0.05, compared with the CI-amidine group mice

FMD exerts robust neuroprotective effects through promoting mitochondria biogenesis, reducing oxidative stress and neuroinflammation [30]. We then explored whether FMD alleviated neuroinflammation via inhibiting NETs production by neutrophils (Fig. 5A). The PWT and PWL of FMD-treated CFA mice were significantly higher than those of CFA mice fed with a normal diet, an effect similar to the analgesic effect of CI-amidine (Fig. 5B, C). Moreover, the combined application of FMD and CI-amidine had a synergistic analgesic effect, whereas the analgesic effect of FMD was reversed by the NETs inducer PMA (Fig. 5B, C). Western blot and immunofluorescence analyses of spinal cord samples showed that FMD reduced MPO and citH3 levels (important NETs components) compared to the control group (Fig. 5D–F). Moreover, compared to the FMD group, the expressions of MPO and citH3 in the FMD + CI-amidine group were significantly more decreased, whereas pretreatment with PMA significantly inhibited the FMD-induced NETs reduction (Fig. 5D–F). These data suggest that FMD relieves allodynia, in part by inhibiting NETs production.

**Fig. 6:**
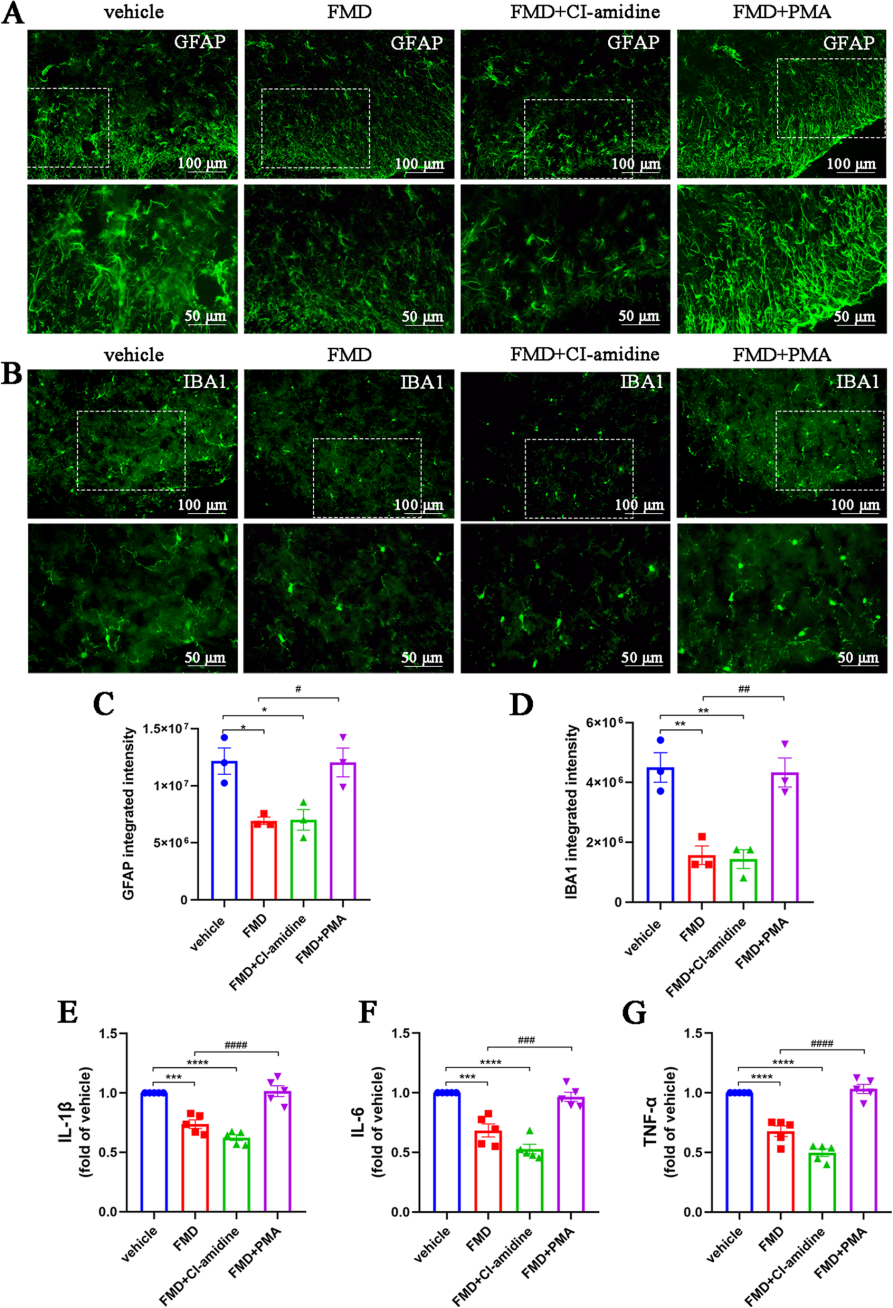
FMD attenuates CFA-induced neuroinflammation by inhibiting the formation of NETs. **A** and **C**. Representative images and quantification of GFAP^+^ astrocytes in spinal cord sections (*n* = 3, F_3, 8_ = 9.14, *P* = 0.0058, scale bars: 50 or 100 μm). **B** and **D**. Representative images and quantification of IBA1^+^ microglia in spinal cord sections (*n* = 3, F_3, 8_ = 16.83, *P* = 0.0008, scale bars: 50 or 100 μm). **E–G**. ELISAs detecting IL-1β, IL-6, and TNF-α in the spinal cords (*n* = 5, IL-1β: F_3, 16_ = 38.3, *P* < 0.0001; IL-6: F_3, 16_ = 34.15, *P* < 0.0001; TNF-α: F_3, 16_ = 61.47, *P* < 0.0001). The expression in the vehicle group was set to 1 for quantification. *P < 0.05, **P < 0.01, ***P < 0.001, ****P < 0.0001, compared with the vehicle group mice. #*P* < 0.05, compared with the FMD group mice

We also explored the effects of FMD on neuroinflammation in the spinal cords of CFA mice. Compared to the normal diet + vehicle group, the numbers of activated astrocytes and microglia in the spinal dorsal horns of mice were significantly decreased in the FMD and FMD + CI-amidine groups, so were the IL-1β, IL-6, and TNF-α levels (Fig. 6A–G). However, compared to FMD alone, pretreatment with PMA significantly reversed the effects of FMD on reducing neuroinflammation (Fig. 6A–G). These results suggest that FMD relieves inflammatory pain by reducing neuroinflammation, mediated by inhibition of NETs formation.

### MAO-B plays a vital role in inducing NETs formation and promoting neuroinflammation

**Fig. 7:**
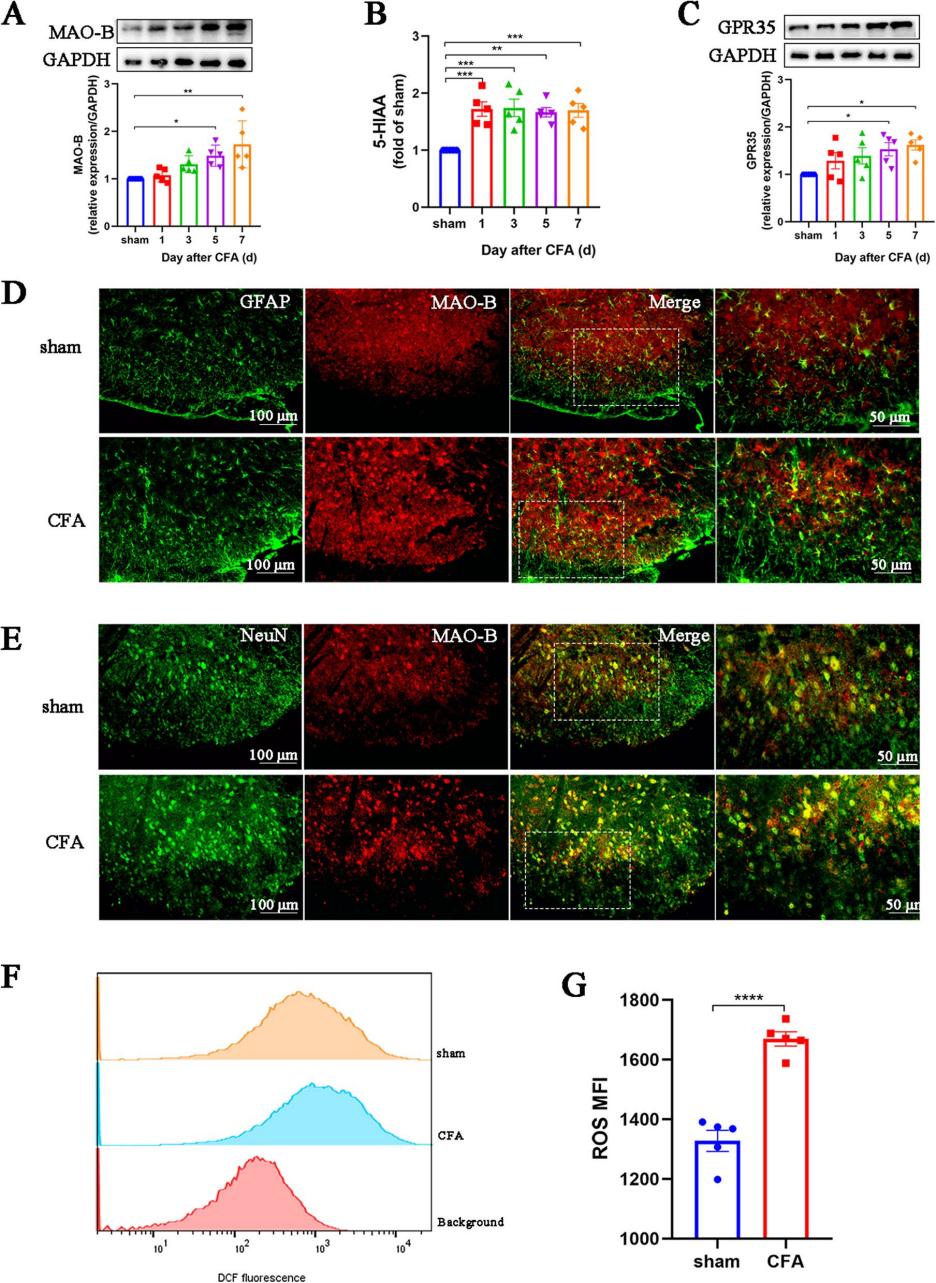
MAO-B plays a vital role in inducing NETs formation and promoting neuroinflammation. **A**. Representative blots and quantification of MAO-B in the spinal cords of sham and CFA mice (*n* = 5, F_4, 20_ = 6.445, *P* = 0.0017). **B**. ELISA detecting 5-HIAA in the spinal dorsal horns (*n* = 5, F_4, 20_ = 8.389, *P* = 0.0004). **C**. Representative blots and quantification of GPR35 in the spinal cords of sham and CFA groups (*n* = 5, F_4, 20_ = 3.227, *P* = 0.0338). **D–E**. Double immunofluorescence staining for MAO-B (red) and GFAP (green) or NeuN (green) in spinal cords (*n* = 3, scale bars: 50 or 100 μm). **F** and **G**. ROS production in the spinal dorsal horns assessed by flow cytometry using DCFH-DA (*n* = 5). *P < 0.05, **P < 0.01, ***P < 0.001, ****P < 0.0001, compared with the sham mice

We then further investigated the mechanisms underlying the FMD-induced inhibition of NETs production. Recent studies have shown that 5-HIAA/GPR35 is a key pathway promoting neutrophil migration to inflammatory sites [27] and that ROS are key factors stimulating neutrophils to produce NETs [31]. Meanwhile, MAO-B is not only a key enzyme that metabolizes serotonin (5-HT) to 5-HIAA but also a major inducer of ROS [27, 32]. Accordingly, we evaluated MAO-B expression in the spinal dorsal horn of CFA mice and found that it increased continuously following CFA administration (Fig. 7A). To determine which cell type expressed the largest amount of MAO-B following CFA treatment, we assessed the cellular localization of MAO-B using immunofluorescence staining. MAO-B was largely expressed by astrocytes (MAO-B^+^ GFAP^+^) and neurons (MAO-B^+^ NeuN^+^; Fig. 7D, E). Additionally, minor colocalization (MAO-B^+^ IBA1^+^) was detected in microglia (Additional file 1). Similar changes were also observed in the 5-HIAA/GPR35 pathway (Fig. 7B, C). Flow cytometry results showed that ROS levels in the spinal cord of mice treated with CFA were markedly higher than those in the sham group (Fig. 7F, G).

### MAO-B inhibition attenuates neuroinflammation and alleviates inflammatory pain by impairing NETs formation

**Fig. 8:**
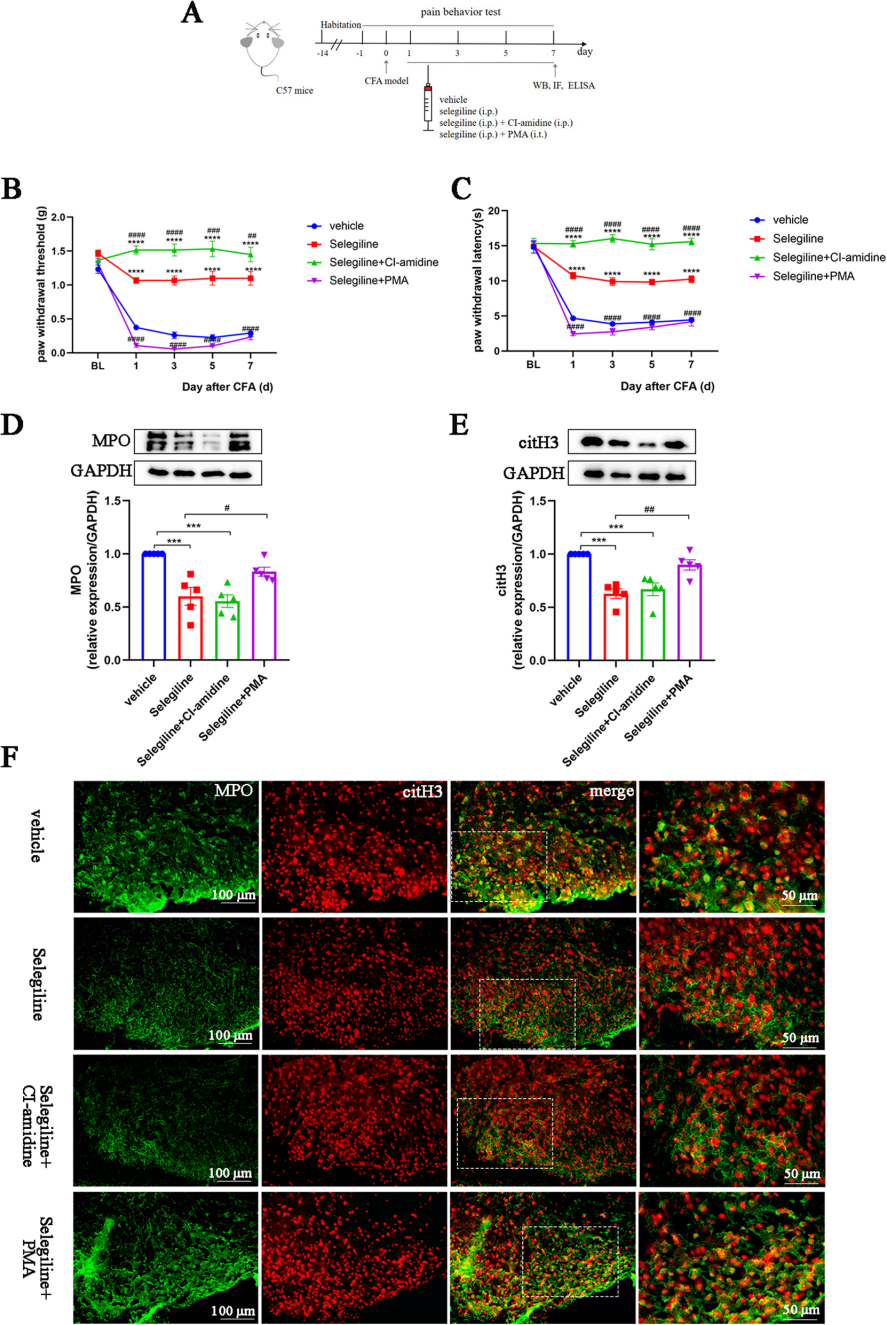
MAO-B inhibition attenuates neuroinflammation and alleviates inflammatory pain by inhibiting NETs formation.** A.** Schematic of the experimental timeline for analyzing the effects of the MAO-B inhibitor selegiline on CFA-induced allodynia and NETs formation. **B.** Paw withdrawal threshold of mice in each group (*n* = 6, group: F‍‍_3,‍‍ ‍15_ = 263.1, *P* < 0.0001; time: F_4, 20_ = 40.53, *P* < 0.0001; interaction: F_12, 60_ = 16.36, *P* < 0.0001). **C.** Paw withdrawal latency of mice in each group (*n* = 6, group: F_3, 15_ = 225.4, *P* < 0.0001; time: F_4, 20_ = 240.1, *P* < 0.0001; interaction: F_12, 60_ = 33.24, *P* < 0.0001). **D** and **E**. Representative blots and quantification of MPO and citH3 in the spinal cords of vehicle, selegiline, selegiline + CI-amidine, and selegiline + PMA groups (*n* = 5, MPO: F_3, 16_ = 13.96, *P* < 0.0001; citH3: F_3, 16_ = 16.13, *P* < 0.0001). **F**. Double immunofluorescence staining for citH3 (red) and MPO (green) in the spinal cords. NETs visualized by colocalization of citH3 and MPO staining (merged images; *n* = 3, scale bars: 50 or 100 μm). *P < 0.05, **P < 0.01, ***P < 0.001, ****P < 0.0001, compared with the vehicle group mice. #*P* < 0.05, compared with the selegiline group mice

**Fig. 9:**
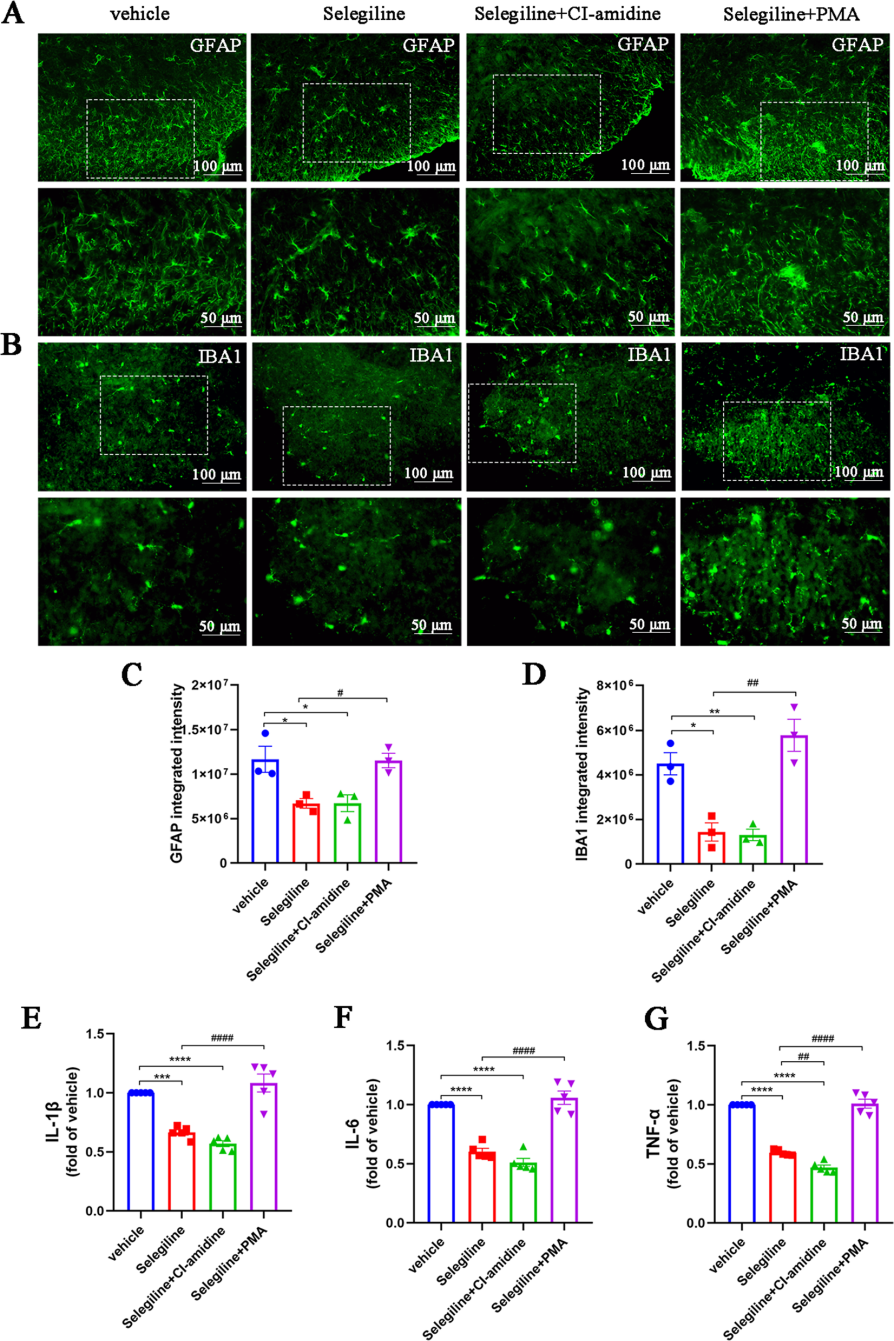
MAO-B inhibition alleviates CFA-induced neuroinflammation by inhibiting NET formation. **A** and **C**. Representative images and quantification of GFAP^+^ astrocytes in spinal cord sections (*n* = 3, F_3, 8_ = 7.936, *P* = 0.0088, scale bars: 50 or 100 μm). **B** and **D**. Representative images and quantification of IBA1^+^ microglia in spinal cord sections (*n* = 3, F_3, 8_ = 20.09, *P* = 0.0004, scale bars: 50 or 100 μm). **E–G**. ELISAs detecting IL-1β, IL-6, and TNF-α in the spinal cords (*n* = 5, IL-1β: F_3, 16_ = 35.98, *P* < 0.0001; IL-6: F_3, 16_ = 58.79, *P* < 0.0001; TNF-α: F_3, 16_ = 155.5, *P* < 0.0001). The expression levels in the vehicle group were set to 1 for quantification. *P < 0.05, **P < 0.01, ***P < 0.001, ****P < 0.0001, compared with the vehicle group mice. #*P* < 0.05, compared with the selegiline group mice

Next, we injected the MAO-B inhibitor selegiline together with the NETs inhibitor CI-amidine or the NETs inducer PMA into CFA mice (Fig. 8A). Compared to the vehicle-treated group, selegiline increased the PWT and PWL of CFA mice; this effect was enhanced by coadministration with CI-amidine. However, PMA reversed the analgesic effect of selegiline (Fig. 8B, C). Consistent with these findings, PMA also abolished the selegiline-induced reduction in MPO and citH3 expression (Fig. 8D–F). Similarly, immunofluorescence and ELISA results showed that selegiline alone and in combination with CI-amidine significantly reduced neuroinflammation in the spinal cords of mice treated with CFA, as characterized by decreases in the numbers of activated astrocytes and microglia and in the levels of IL-1β, IL-6, and TNF-α. However, PMA reversed the anti-neuroinflammatory effects of selegiline (Fig. 9A–G). These results suggest that MAO-B/5-HIAA/GPR35 and MAO-B/ROS signaling pathways exacerbate neuroinflammation, as well as mechanical and thermal allodynia, via induction of NETs formation and may be vital downstream targets of FMD-induced inhibition of NETs.

### Specific knockdown of neuron and astrocyte MAO-B alleviates inflammatory pain

**Fig. 10:**
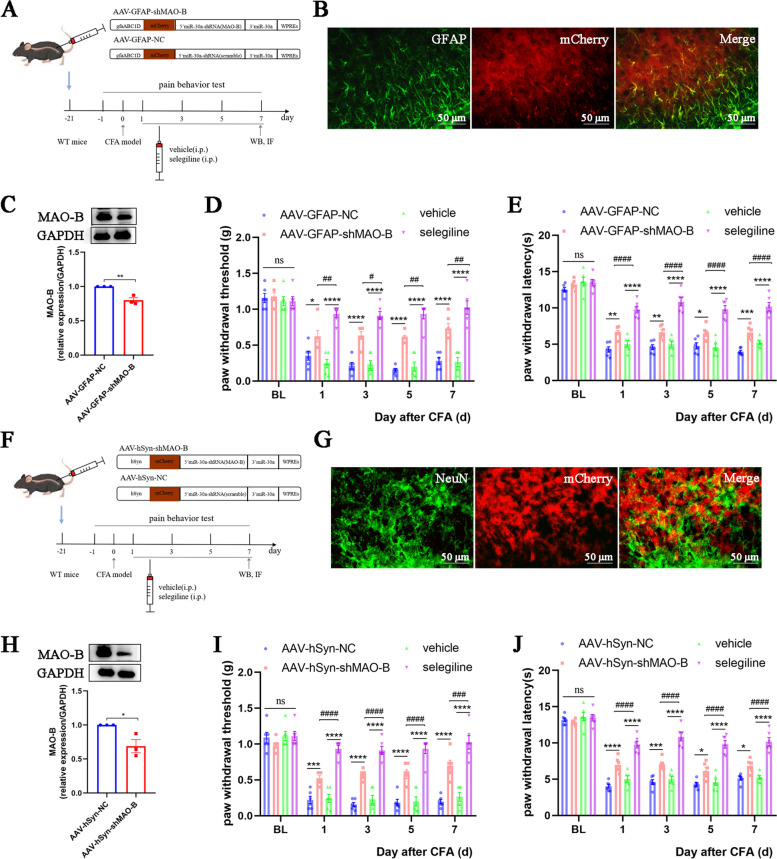
Specific knockdown of neuron and astrocyte MAO-B alleviates inflammatory pain. **A**. Schematic paradigm of MAO-B knockdown in spinal cord astrocytes of CFA mice.** B**. Fluorescence images of viral particles in the spinal cord of CFA mice treated with AAV-GFAP-shMAO-B virus (*n* = 3, scale bars: 50 μm).** C**. Representative blots and quantification of MAO-B in the spinal cords of mice (*n* = 3).** D**. Paw withdrawal threshold of mice in each group (*n* = 6, group: F_3, 15_ = 69.43, *P* < 0.0001; time: F_4, 20_ = 116.6, *P* < 0.0001; interaction: F_12, 60_ = 9.147, *P* < 0.0001). **E**. Paw withdrawal latency of mice in each group (*n* = 6, group: F_3, 15_ = 107.5, *P* < 0.0001; time: F_4, 20_ = 304, *P* < 0.0001; interaction: F_12, 60_ = 7.103, *P* < 0.0001). **F**. Schematic paradigm of MAO-B knockdown in spinal cord neurons of CFA mice. **G**. Fluorescence images of viral particles in the spinal cord of CFA mice treated with AAV-hSyn-shMAO-B virus (*n* = 3, scale bars: 50 μm). **H**. Representative blots and quantification of MAO-B in the spinal cords of mice (*n* = 3). **I**. Paw withdrawal threshold of mice in each group (*n* = 6, group: F_3, 15_ = 57.55, *P* < 0.0001; time: F_4, 20_ = 117.5, *P* < 0.0001; interaction: F_12, 60_ = 11.5, *P* < 0.0001). **J**. Paw withdrawal latency of mice in each group (*n* = 6, group: F_3, 15_ = 138.3, *P* < 0.0001; time: F_4, 20_ = 227.5, *P* < 0.0001; interaction: F_12, 60_ = 8.401, *P* < 0.0001). *P < 0.05, **P < 0.01, ***P < 0.001, ****P < 0.0001, compared with the AAV-NC or vehicle group mice. #*P* < 0.05, compared with the selegiline group mice

To further determine the role of astrocyte and neuron MAO-B in pain, we transfected spinal cord astrocytes and neurons with RNA interference viruses driven by gfaABC1D and hSyn promoters to knock down MAO-B (Fig. 10A, F). The western blot and double immunofluorescence staining results confirmed the cell type-specific knockdown of MAO-B (Fig. 10 B, C, G, H). Behavioral results showed that specific knockdown of astrocyte and neuron MAO-B alleviated mechanical and thermal allodynia in CFA mice. However, intraperitoneal injection of the MAO-B inhibitor (selegiline) elicited a more effective analgesic effect than specific MAO-B knockdown in neurons or astrocytes (Fig. 10 D, E, I, J). Hence, it is suggested that astrocyte and neuron MAO-B have equally important roles in regulating hyperalgesia. Accordingly, selegiline was employed in subsequent experiments to inhibit MAO-B expression.

### MAO-B/5-HIAA/GPR35 and MAO-B/ROS signaling pathways are essential for FMD-induced NETs reduction and pain relief

**Fig. 11:**
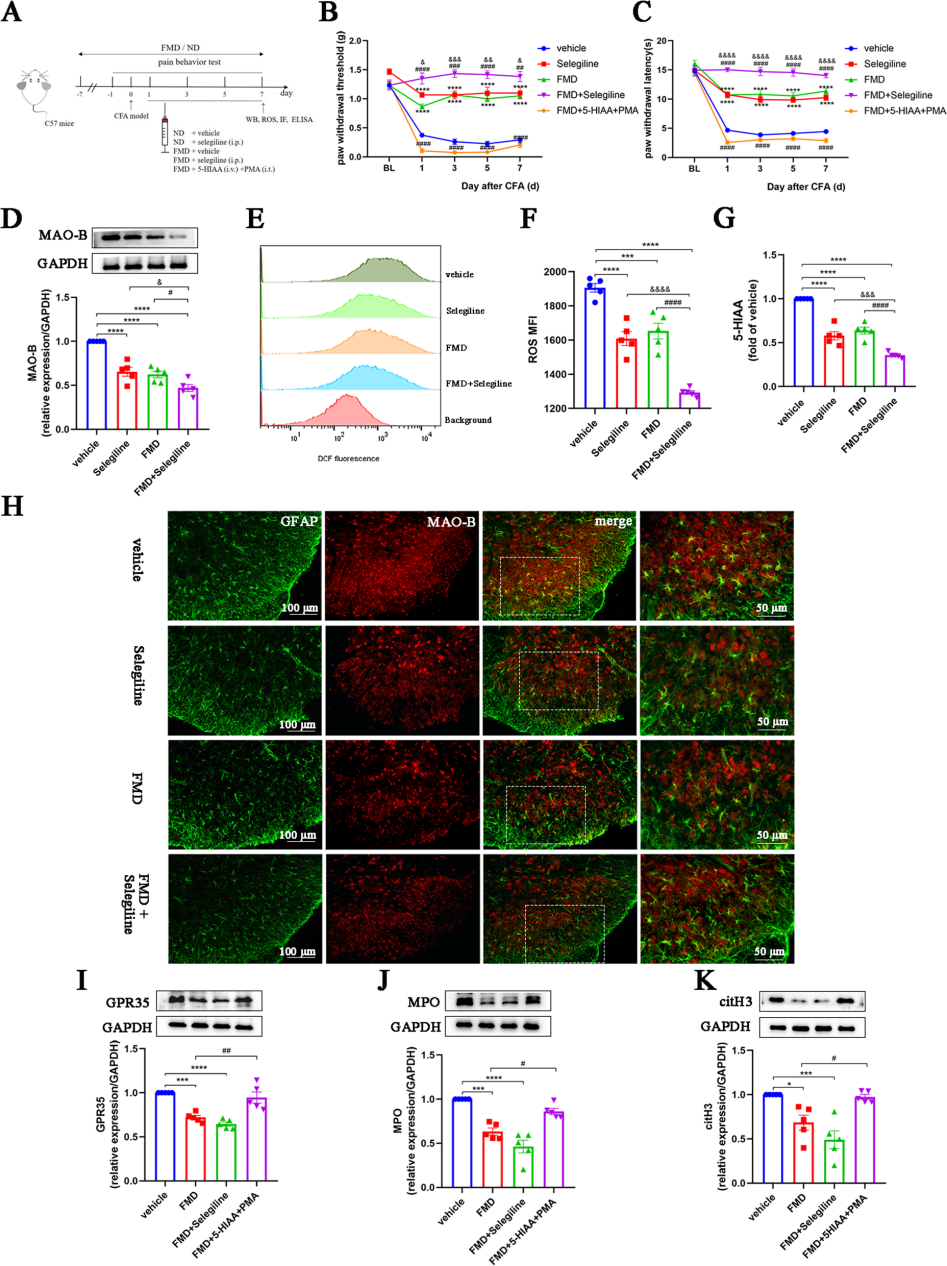
The MAO-B/5-HIAA/GPR35 and MAO-B/ROS pathways are essential for FMD-induced NETs reduction and pain relief. **A**. Schematic of the experimental timeline to explore the necessity of MAO-B and its downstream products for FMD-induced NETs reduction and pain relief. **B**. Paw withdrawal threshold of mice (*n* = 6, group: F_4, 20_ = 152, *P* < 0.0001; time: F_4, 20_ = 143.7, *P* < 0.0001; interaction: F_16, 80_ = 16.33, *P* < 0.0001). **C**. Paw withdrawal latency of mice (*n* = 6, group: F_4, 20_ = 193.8, *P* < 0.0001; time: F_4, 20_ = 331.4, *P* < 0.0001; interaction: F_16, 80_ = 29.24, *P* < 0.0001). **D**. Representative blots and quantification of MAO-B in the spinal cords (*n* = 5, F_3, 16_ = 35.77, *P* < 0.0001). **E** and **F**. ROS production in the spinal dorsal horns assessed by flow cytometry using DCFH-DA (*n* = 5, F_3, 16_ = 56.4, *P* < 0.0001). **G**. ELISA detection of 5-HIAA in the spinal dorsal horns (*n* = 5, F_3, 16_ = 72.25, *P* < 0.0001). **H**. Double immunofluorescence staining for MAO-B (red) and GFAP (green) in the spinal cords (*n* = 3, scale bars: 50 or 100 μm). **I**. Representative blots and quantification of GPR35 in the spinal cords of vehicle, FMD, FMD + selegiline, and FMD + 5-HIAA + PMA groups (*n* = 5, F_3, 16_ = 23.56, *P* < 0.0001). **J** and **K**. Representative blots and quantification of MPO and citH3 in the spinal cords of vehicle, FMD, FMD + selegiline, and FMD + 5-HIAA + PMA groups (*n* = 5, MPO: F_3, 16_ = 28.35, *P* < 0.0001; citH3: F_3, 16_ = 13.39, *P* = 0.0001). *P < 0.05, **P < 0.01, ***P < 0.001, ****P < 0.0001, compared with the vehicle group mice. #*P* < 0.05, compared with the FMD group mice. &*P* < 0.05, compared with the selegiline group mice

To further investigate whether FMD relieves NETs-related neuroinflammation and pain through the MAO-B/5-HIAA/GPR35 and MAO-B/ROS pathways, we compared CFA mice exposed to FMD, selegiline, or FMD combined with selegiline (Fig. 11A). Compared to CFA mice with a normal diet and vehicle treatment, the PWT and PWL in the selegiline and FMD groups were increased. Moreover, FMD and selegiline exhibited synergistic analgesic effects (Fig. 11B, C). Western blot results indicated that the expression of MAO-B in the selegiline and FMD groups was markedly decreased; combined FMD and selegiline significantly decreased these levels compared with either treatment alone (Fig. 11D). Immunofluorescence colocalization results showed that MAO-B expression in astrocytes and neurons in the spinal dorsal horn of mice treated with CFA was significantly decreased by FMD and selegiline treatment (Fig. 11H and Additional file 2). Compared to the control group, the ROS and 5-HIAA levels in the spinal cords of CFA mice treated with FMD or selegiline alone were significantly decreased, while the combined treatment was more effective (Fig. 11E–G).

Considering that NETs formation requires recruitment of neutrophils to the spinal cord and NETs induction, we supplemented FMD-treated mice with MAO-B downstream product 5-HIAA and NETs inducer PMA to investigate whether the analgesic effect of FMD was blocked (Fig. 11A). The analgesic effect of FMD was blocked by the supplementation of 5-HIAA and PMA (Fig, 11B, C). Consistent with the behavioral data, western blotting showed that GPR35, MPO, and citH3 levels substantially decreased in the FMD and FMD + selegiline groups; however, the FMD-induced GPR35 and NETs reduction was abolished by 5-HIAA and PMA co-treatment (Fig. 11I–K). These data suggest that the MAO-B/5-HIAA/GPR35 and MAO-B/ROS pathways are essential for FMD-induced NETs reduction and pain relief.

### MAO-B/5-HIAA/GPR35 and MAO-B/ROS pathways are essential for FMD-induced inhibition of neuroinflammation

**Fig. 12:**
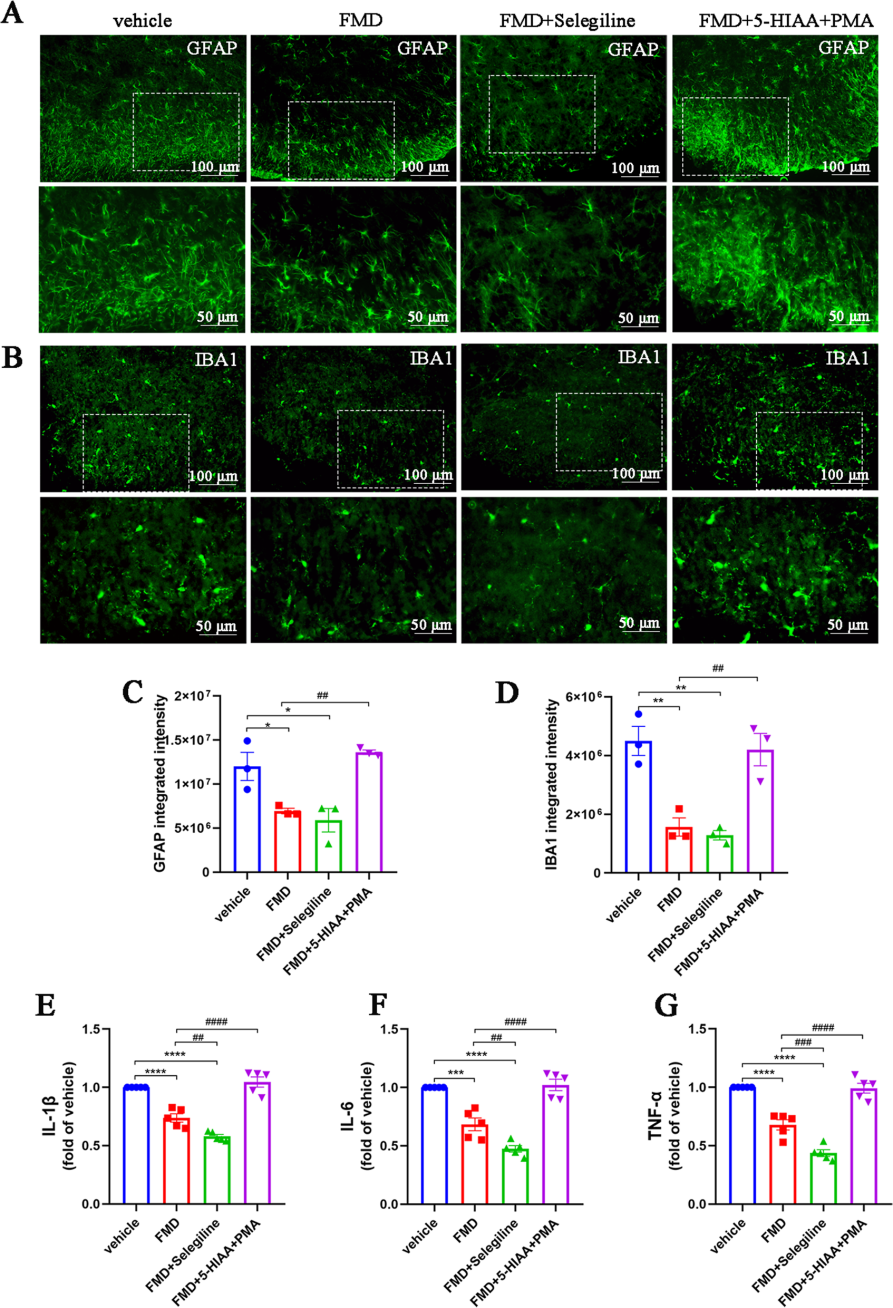
The MAO-B/5-HIAA/GPR35 and MAO-B/ROS pathways are essential for FMD-induced neuroinflammation reduction. **A** and **C**. Representative images and quantification of GFAP^+^ astrocytes in spinal cord sections (*n* = 3, F_3, 8_ = 12.71, *P* = 0.0021, scale bars: 50 or 100 μm). **B** and **D**. Representative images and quantification of IBA1^+^ microglia in spinal cord sections (*n* = 3, F_3, 8_ = 17.11, *P* = 0.0008, scale bars: 50 or 100 μm). **E–G**. ELISAs detecting IL-1β, IL-6, and TNF-α in the spinal cords of mice (*n* = 5, IL-1β: F_3, 16_ = 56.48, *P* < 0.0001; IL-6: F_3, 16_ = 45.68, *P* < 0.0001; TNF-α: F‍‍_3, ‍16_ = 65.76, *P* < 0.0001). The expression levels in the vehicle group were set to 1 for quantification. *P < 0.05, **P < 0.01, ***P < 0.001, ****P < 0.0001, compared with the vehicle group mice. #*P* < 0.05, compared with the FMD mice

Finally, we explored the role of MAO-B in the inhibition of FMD-induced neuroinflammation. Both FMD alone and in combination with selegiline significantly reduced the numbers of activated astrocytes and microglia (Fig. 12A–D). However, the combined treatment had no synergistic effect, while the combined application of 5-HIAA and PMA blocked the FMD effect. Similarly, FMD alone or in combination with selegiline decreased the levels of IL-1β, IL-6, and TNF-α; however, this effect was eliminated by 5-HIAA and PMA co-pretreatment (Fig. 12E–G). These data suggest that the MAO-B/5-HIAA/GPR35 and MAO-B/ROS pathways are essential for FMD-induced inhibition of neuroinflammation.

## Discussion

**Fig. 13:**
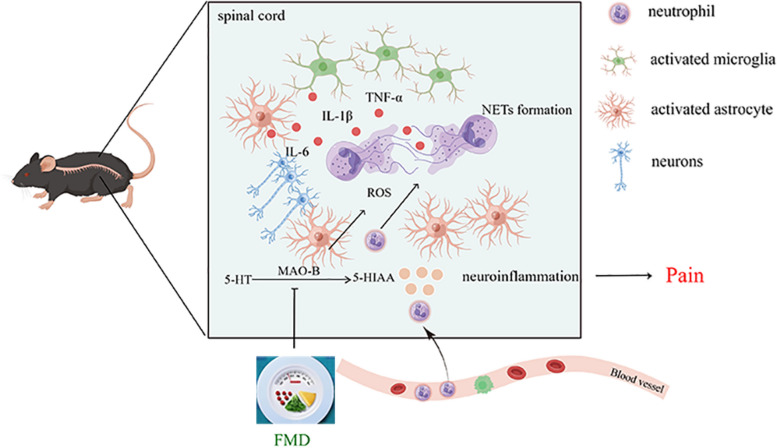
Model showing the mechanism of FMD inhibiting NETs and relieving pain. NETs released by neutrophils in the spinal cord promote the development of pain by aggravating neuroinflammation. FMD inhibits both NETs formation and NETs-related neuroinflammation by downregulating MAO-B/5-HIAA/GPR35 and MAO-B/ROS pathways of astrocytes and neurons in the spinal cords of mice. FMD: fasting-mimicking diet

Chronic pain is common and not only exerts great unpleasant physiological effects on patients but also leads to emotional disorders, as well as huge social and economic burdens [33]. As it is widely recognized that chronicity of neuroinflammation is the primary cause of chronic pain [4], early intervention for acute pain is effective in preventing persistent pain [2]. Our results indicate that NETs generated by neutrophils in the spinal cord promote the development of acute pain by exacerbating neuroinflammation. FMD effectively inhibited the progress of pain and enhanced the analgesic effects of NETs inhibitors. Furthermore, FMD inhibited the formation of NETs and NETs-related neuroinflammation by downregulating the MAO-B/5-HIAA/GPR35 and MAO-B/ROS pathways in astrocytes and neurons within the spinal cords of mice. In particular, MAO-B is a potential mediator of neuron and astrocyte–neutrophil crosstalk (Fig. 13).

There is widespread interest in the communication between immune and nerve cells and its role in pain. In neuropathic pain models, the apparent infiltration of immune cells into the CNS is attributed to disruption of the blood–brain barrier or an increase in its permeability [34–36]. Neutrophils infiltrating the CNS elicit neuroinflammation and central sensitization by releasing proinflammatory mediators [4]. Consistently, we observed an increase in BSCB permeability and a significant augmentation of neutrophil infiltration in the spinal cords of mice in the inflammatory pain model. Upon activation, neutrophils release NETs, which participate in the progression of several neurological diseases, including multiple sclerosis, Alzheimer's disease, and ischemic stroke through inducing neuroinflammation and neurotoxicity [37, 38]. Here, we found that large amounts of NETs (citH3^+^ MPO^+^) accumulated in the spinal dorsal horn during the acute and subacute phases of inflammatory pain (Days 1–7). Following administration of PAD4 siRNA or CI-amidine to inhibit NETs formation, mechanical and thermal allodynia in CFA mice were alleviated, indicating that NETs in the spinal cord participated in pain progression.

Neuroinflammation, distinguished by immune cell infiltration, glial cell activation, and the generation of inflammatory mediators in the CNS, is crucial in the onset and persistence of pain [4, 39]. NETs inhibitors exert anti-inflammatory and microglia-inhibiting effects in animal models of multiple sclerosis and neonatal hypoxia–ischemia [40, 41]. Consistently, we found that astrocytes and microglia in the spinal cords of CFA mice were significantly activated, accompanied by marked elevation of IL-1β, IL-6, and TNF-α. These responses were completely inhibited by the NETs inhibitor CI-amidine. This suggests that NETs promote the development of inflammatory pain, at least in part, by aggravating neuroinflammation.

Increasing evidence suggests that fasting holds significant promise in treating diverse chronic conditions, especially neurological disorders [30]. Intermittent fasting confers neuroprotection in rats with intracerebral hemorrhage by attenuating excessive neuroinflammatory responses and neurological dysfunction, as well as inhibiting neuronal apoptosis [42]. Furthermore, while several clinical studies have shown the effectiveness of caloric restriction in alleviating osteoarthritis-related pain, there is currently no consensus on the short-term and long-term impacts of fasting on pain, and the underlying mechanism(s) remain unclear [18, 43, 44]. Here, we demonstrated that FMD generates broad positive effects in CFA mice, including alleviation of neuroinflammation, inhibition of neutrophil release of NETs, and attenuation of inflammatory pain. The safety of FMD has also been evaluated in other models, showing no significant adverse effects, thus suggesting its potential clinical utility in the treatment of inflammatory pain [14, 45]. Nevertheless, further research is warranted to evaluate the analgesic effects of FMD in diverse populations, encompassing different age groups and sexes. A previous study reported that long-term caloric restriction had an antinociceptive effect in only adult mice, while this effect was weak or even absent in aging mice [46]. Boccella et al. reported that a short-term starvation protocol effectively reduced chronic pain onset more in female than male mice, which was attributed to the liver-produced ketone body, β-hydroxybutyrate (BHB) [47]. The analgesic effects of fasting and BHB on chronic pain resulting from nerve injury are mediated through the BHB receptor GPR109A (also known as hydroxycarboxylic acid receptor 2 (HCAR2)) [47]. GPR109A has garnered increasing attention in recent years as a crucial target for the regulation of neuroinflammation and pain [48, 49]. However, whether FMD-mediated inhibition of neuroinflammation and inflammatory pain is dependent on BHB/GPR109A in male mice remains to be further explored. Recent studies indicate that HCAR2 has neuroprotective and anti-inflammatory properties, achieved through the promotion of microglia transformation to an anti-inflammatory phenotype or by inhibiting microglia activation and the release of pro-inflammatory factors [50, 51]. HCAR2 regulates the phenotypic transition of microglia/macrophages towards the protective phenotype, promoting effective phagocytosis of myelin debris and remyelination [52, 53]. The regulatory effects of HCAR2 on microglia and other immune cells in various physiological conditions suggest the potential role of HCAR2 in the neuroimmune mechanisms underlying pain modulation during FMD.

Based on these results, we further investigated the mechanism by which FMD inhibits NETs formation. A recent study showed that 5-HIAA, a metabolite of 5-HT, promotes the rapid recruitment of neutrophils to inflammatory sites by binding to GPR35 receptors on neutrophils [27]. Moreover, GPR35 in the dorsal root ganglion and spinal cord is an important therapeutic target for neuropathic and inflammatory pain [54, 55]. ROS is a key inducer of NETs production by neutrophils and is involved in many types of pain [31, 56, 57]. Interestingly, MAO-B is not only a key enzyme that promotes the metabolism of 5-HT to 5-HIAA [27] but also a primary source of ROS production [32, 58]. As expected, the expression of MAO-B and its downstream products 5-HIAA, as well as ROS were significantly upregulated in the spinal dorsal corn of CFA mice. Specific knockdown of MAO-B in astrocytes and neurons or pharmacological inhibition of MAO-B expression effectively relieved inflammatory pain. Furthermore, the inducer of NETs, PMA, reversed the analgesic and anti-inflammatory effects of selegiline. Therefore, we conclude that MAO-B promotes pain development by inducing NETs formation. Consistent with a previous finding that fasting inhibits MAO activity in the white adipose tissue of rats [59], we found that FMD pretreatment inhibited the expression of MAO-B in neurons and astrocytes. Finally, through a rescue experiment, we proved that the MAO-B/5-HIAA/GPR35 and MAO-B/ROS signaling pathways are required for FMD-induced NETs reduction and pain relief.

In addition, we found that MAO-B may function as a mediator for the crosstalk between nerve and immune cells. Indeed, complex bidirectional communication occurs between the nervous and immune systems [60]. Upon nerve tissue injury, immune cells such as T cells, macrophages, and neutrophils are recruited by nerve cells to the affected tissue, leading to neuroinflammation [4, 61]. In the current study, immunofluorescence colocalization staining showed that after CFA administration, astrocytes and neurons in the spinal dorsal horn expressed large amounts of MAO-B, whereas microglia expressed less of this enzyme. However, specific knockdown of neuron and astrocyte MAO-B alleviates inflammatory pain. Following FMD or treatment with an MAO-B inhibitor, the numbers of MAO-B-expressing astrocytes and neurons in the spinal cords of CFA mice were significantly decreased. These results suggest that in the mouse model of CFA-induced inflammatory pain, MAO-B expressed by astrocytes and neurons promotes neutrophil migration to the spinal cord and the subsequent release of NETs, which further promotes the activation of astrocytes and microglia, leading to a cascade amplification of inflammation. FMD blocks this amplification progress by inhibiting MAO-B. The recent reports support our results, which show that astrocytes respond quickly after injury, secrete many soluble molecules, and recruit neutrophils and other immune cells to aggravate inflammation and worsen functional recovery [62, 63]. However, considering the FMD-mediated inhibition of NETs formation, the complex mechanism of crosstalk between the nervous and immune systems requires further elucidation, which are issues we plan to focus on in the future.

Our study has several limitations. Although our results reveal the contribution of crosstalk between neutrophils and central immune cells (microglia and astrocytes) to the development of inflammatory pain in male C57 mice, it should not be disregarded that the neuroimmune mechanisms involved in abnormal pain differ between males and females [64, 65]. Recent evidence suggests that hyperalgesia in male mice depends on the regulation of microglia [66]. Specifically, toll-like receptor 4 and purinergic P2X4 receptors, which are selectively expressed on microglia, are essential for mediating hyperalgesia in male but not female mice [67]. Moreover, Vegeto et al. found that estrogen inhibits the pro-inflammatory phenotypic transformation of microglia induced by lipopolysaccharide [68]. Female mice achieve hyperalgesia preferentially through T cells and switch to glial cell-dependent pathway when adaptive immune cells are deficient [66]. Recent evidence shows that the level of T cell markers in the spinal cord of CD-1 mice is significantly higher for female than male mice 7 days after nerve injury [66]. Female T-cells have an inherent tendency to proliferate and produce higher levels of interferon-γ, while exhibiting lower levels of IL-17A compared to male T-cells, which was associated with androgen, particularly testosterone [69]. Although both cytokines have been implicated in pain, T-cells infiltrating the spinal cord following nerve injury mainly exhibit the Th1 subtype (secreting interferon-γ) [70]. Hence, infiltrating T-cells in the spinal cord of male mice might have a limited role in mediating abnormal pain, potentially indicating the involvement of alternative immune pathways such as microglia [66]. These results reveal significant sex disparities in neuroimmune mechanisms concerning chronic pain, yet a precise comprehension of neuroimmune regulation in both males and females remains elusive. Our research necessitates further validation in female mice.

## Conclusions

In summary, the current findings demonstrate that NETs released by neutrophils in the spinal cord participate in the development of acute pain. FMD prevents the development of acute pain by inhibiting NETs formation and neuroinflammation. We also found that MAO-B and its downstream 5-HIAA/GPR35 and ROS pathways are important FMD targets that inhibit NETs formation and relieve neuroinflammation. Hence, this study identified new targets for pain prevention and treatment.

### Supplementary Information

**Additional file 1. **MAO-B was rarely expressed by microglia in the spinal cords of CFA mice. Double immunofluorescence staining for MAO-B (red) and IBA1 (green) in a spinal cord section (*n* = 3, scale bars: 50 or 100 μm).

**Additional file 2.** MAO-B abundance is decreased in the spinal cord neurons of CFA mice after FMD and selegiline treatment. Double immunofluorescence staining for MAO-B (red) and NeuN (green) in a spinal cord section (*n* = 3, scale bars: 50 or 100 μm).

## Data Availability

All data generated or analyzed during this study are included in this article (and its additional files).

## Abbreviations

5-HIAA: 5-Hydroxyindoleacetic acid
BHB: β-Hydroxybutyrate
BSCB: Blood–spinal cord barrier
CFA: Complete Freund's adjuvant
CNS: Central nervous system
FMD: Fasting-mimicking diet
GFAP: Glial fibrillary acidic protein
GPR35: G-protein-coupled receptor 35
HCAR2: Hydroxycarboxylic acid receptor 2
IBA1: Ionized calcium binding adaptor molecule 1
i.p.: Intraperitoneal injection
i.t.: Intrathecal injection
MAO-B: Monoamine oxidase-B
MFI: Mean fluorescence intensity
MPO: Myeloperoxidase
NETs: Neutrophil extracellular traps
PBS: Phosphate-buffered saline
PMA: Phorbol 12-myristate 13-acetate
ROS: Reactive oxygen species
TLR4: Toll-like receptor 4
TNF-α: Tumor necrosis factor-alpha
