# Supplementary figures and images for: Fasting-mimicking diet alleviates inflammatory pain by inhibiting neutrophil extracellular traps formation and neuroinflammation in the spinal cord
Source: Cell Commun Signal. 2023 Sep 21;21:250. doi: 10.1186/s12964-023-01258-2 (PMC10512659; doi:10.1186/s12964-023-01258-2)

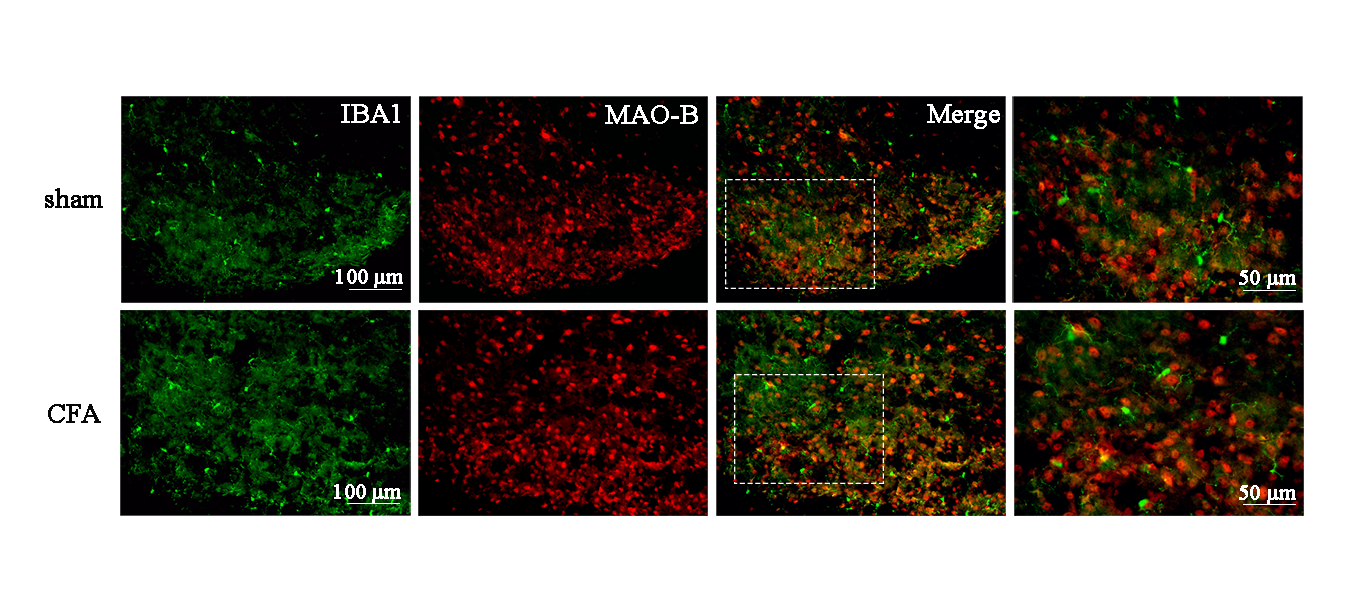

Supplement: Supplementary file 2 — Additional file 1. MAO-B was rarely expressed by microglia in the spinal cords of CFA mice. Double immunofluorescence staining for MAO-B (red) and IBA1 (green) in a spinal cord section (n = 3, scale bars: 50 or 100 μm). [file 12964_2023_1258_MOESM1_ESM.tif]

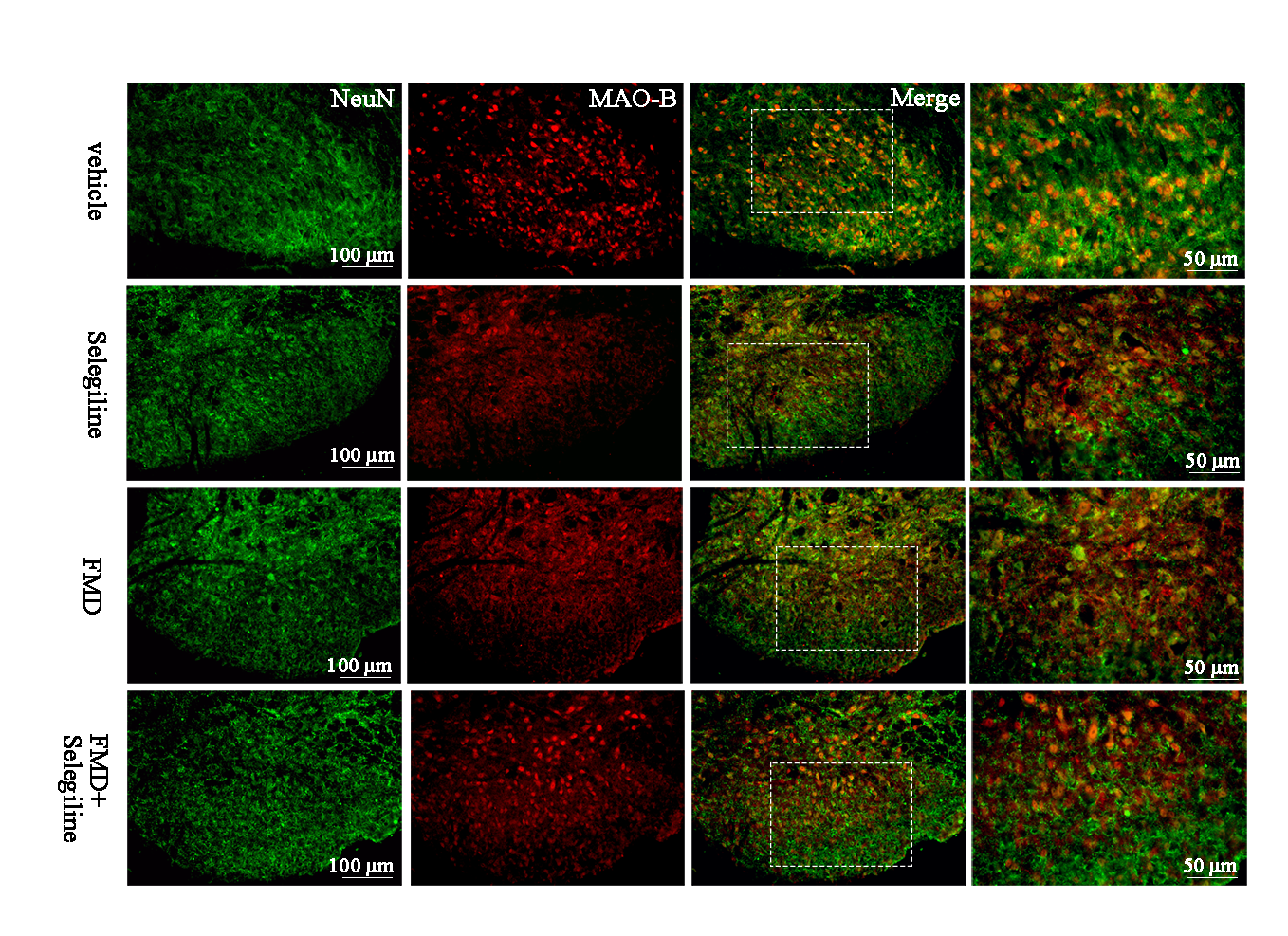

Supplement: Supplementary file 3 — Additional file 2. MAO-B abundance is decreased in the spinal cord neurons of CFA mice after FMD and selegiline treatment. Double immunofluorescence staining for MAO-B (red) and NeuN (green) in a spinal cord section (n = 3, scale bars: 50 or 100 μm). [file 12964_2023_1258_MOESM2_ESM.tif]
